# Food allergy outside the eight big foods in Europe: A systematic review and meta‐analysis

**DOI:** 10.1002/clt2.12338

**Published:** 2024-02-07

**Authors:** Giulia C. I. Spolidoro, Sungkutu Nyassi, Daniil Lisik, Athina Ioannidou, Mohamed Mustafa Ali, Yohannes Tesfaye Amera, Graciela Rovner, Ekaterina Khaleva, Carina Venter, Ronald van Ree, Margitta Worm, Berber Vlieg‐Boerstra, Aziz Sheikh, Antonella Muraro, Graham Roberts, Bright I. Nwaru

**Affiliations:** ^1^ Department of Clinical Sciences and Community Health University of Milan Milan Italy; ^2^ Krefting Research Centre University of Gothenburg Gothenburg Sweden; ^3^ School of Public Health and Community Medicine Institute of Medicine University of Gothenburg Gothenburg Sweden; ^4^ ACT Institutet Sweden Vejbystrand Sweden; ^5^ Division of Physiotherapy Department of Neurobiology Care Sciences and Society Karolinska Institutet Stockholm Sweden; ^6^ Faculty of Medicine University of Southampton Southampton UK; ^7^ Section of Allergy & Immunology School of Medicine University of Colorado Denver Children's Hospital Colorado Aurora Colorado USA; ^8^ Department of Experimental Immunology and Department of Otorhinolaryngology Amsterdam University Medical Centers Amsterdam The Netherlands; ^9^ Division of Allergy and Immunology Department of Dermatology, Allergy and Venerology Charité Universitätsmedizin Berlin Berlin Germany; ^10^ Department of Pediatrics OLVG Hospital Amsterdam The Netherlands; ^11^ Rijnstate Allergy Centre Rijnstate Hospital Arnhem The Netherlands; ^12^ Usher Institute University of Edinburgh Edinburgh UK; ^13^ Department of Mother and Child Health The Referral Centre for Food Allergy Diagnosis and Treatment Veneto Region University of Padua Padua Italy; ^14^ David Hide Asthma and Allergy Centre St Mary's Hospital Isle of Wight UK; ^15^ Wallenberg Centre for Molecular and Translational Medicine University of Gothenburg Gothenburg Sweden

**Keywords:** epidemiology, Europe, food allergy, sensitization, systematic review

## Abstract

**Background:**

The 2014 estimates of prevalence of food allergy (FA) in Europe published by the European Academy of Allergy and Clinical Immunology included only the eight so‐called big foods (cow's milk/egg/wheat/soy/peanut/tree nuts/fish/shellfish). Those estimates have recently been updated. Complementing this, we sought to identify and estimate the prevalence of allergy to other foods that have been reported during the last decade.

**Methods:**

Six databases were searched for studies published 2012–2021. Random‐effects meta‐analysis was performed to derive pooled prevalence of allergy to each food.

**Results:**

Twenty‐seven studies were included, containing a total of 66 FAs. Among the most frequently reported FAs, the lifetime and point prevalence range of self‐reported kiwi allergy was 0.1%–1.0% and 0.2%–8.1%, respectively, while the food challenge (FC)‐verified kiwi allergy point prevalence range was 0.01%–0.10%. The point prevalence range for self‐reported peach allergy was 0.2%–3.2%, while the range for FC‐verified peach allergy was 0.02%–0.05%. The lifetime and point prevalence range for self‐reported tomato allergy was 0.01%–1.8% and 0.2%–2.1%, respectively.

**Conclusion:**

Allergy to some foods traditionally not considered important are now emerging as relevant FAs. The focus on FA in Europe should not be limited to the so‐called eight big FA, but extended to other types of foods which need to be considered both for clinical purposes and population risk assessment.

## 1 INTRODUCTION

Food allergies (FAs) have become a common topic for healthcare systems, as the incidence and prevalence have reportedly increased over the last decades. However, there is a need to improve this evidence base in order to gain a better appreciation of the frequency of FA across Europe, through which its healthcare and societal burden can be elucidated more clearly. A decade ago, the European Academy of Allergy and Clinical Immunology (EAACI) published a systematic review and meta‐analysis on the prevalence of food allergy/sensitization (FA/FS) in Europe based on studies published between 2000 and 2012, but the focus was on allergy to the eight so‐called big foods (i.e., cow's milk, hen's egg, wheat, soy, peanut, tree nuts, fish, and shellfish).^1^, ^2^ We recently published a 10‐year update of that work, which showed that, although the prevalence of any FA indeed increased in the last decade, the prevalence of FAs to the eight big foods did not change as much.^3^, ^4^, ^5^ Indeed, many other foods can elicit FA/FS, including fruit, vegetables/legumes, cereal, meat, and others. An additional objective of our update was to identify and estimate the prevalence of FA/FS in foods other than the eight big foods that have been reported during the last decade. The current work therefore summarizes the available evidence on FA/FS to foods other than the eight so‐called big foods in Europe and estimates their prevalence, where data allow.

## 2 METHODS

### 2.1 Protocol registration, search strategies, and study identification and selection

Before starting the systematic review, a protocol was registered on the International Prospective Register of Systematic Reviews (PROSPERO) (registration number CRD42021266657). The search strategy was adapted from the methodology employed in the previous EAACI systematic review and meta‐analysis.^1^, ^2^ In brief, we combined the two concepts of FA/FS and epidemiology to detect all relevant literature. We searched six electronic databases (MEDLINE, Embase, CINAHL, Web of Science, Cochrane Library, and Scopus) to collect papers and conference abstracts on FA/FS in Europe published between September 2012 and June 2021. We retained all keywords employed in the 2014 reviews; additional keywords were included to avoid missing any relevant studies, as well as to account for developments that have occurred in the chosen databases in the last 10 years. The full search strategy has been reported in the Supporting Information S1 of our previously published paper on the frequency of any FA in Europe.^3^ We did not apply any language restriction to the database search. Studies not written in English were translated by a member of our research team fluent in the language. In case translation was not possible, but an English abstract was available, relevant data were extracted from the paper abstract, while at the same time employing Google Translate to translate the text. Having a clear idea of the paper content from the abstract summary allowed us to limit the risk of data misinterpretation.

All studies that investigated FA/FS in the general European population, of any age and gender, were considered eligible.

The following study types were eligible for inclusion: systematic reviews, cross‐sectional studies, cohort studies, case‐control studies, clinical trials and routine healthcare studies. Narrative review, discussion papers, non‐research letters or editorials, case‐series, case‐studies, and animal studies were excluded. Relevant papers were screened by four independent reviewers, working in pairs (SN/GS and YA/MA), first by title and/or abstract, and later by full text. In case of disagreement between the pairs, conflicts were resolved with consensus or after consulting the project PI (BN). We employed the Preferred Reporting Items for Systematic Reviews and Meta‐Analysis (PRISMA) flow diagram to describe the screening process and we conducted our research according to the guidelines of the PRISMA 2020 Statement.^6^

### 2.2 Outcomes

The systematic review aimed to provide up‐to‐date estimates on the incidence, prevalence, and time trends for FAs outside the eight big FAs in Europe for the period 2012–2021.

An allergy to any food outside the eight big foods were considered in the analysis, including fruits, vegetables, legumes, meat, cereals (not including wheat), seeds, herbs, spices, and condiments, and many other food types. Due to the paucity of data on incidence and time trends, meta‐analysis was performed only on data on lifetime and point prevalence, similar to what was undertaken in the 2014 EAACI review of any FA and on FA to the eight big foods, as well as in the 2022/3 update.^1^, ^2^, ^3^, ^4^, ^5^

We could not differentiate between immunoglobulin E (IgE)‐mediated and non‐IgE‐mediated FA, as this was not usually differentiated in the included studies.

The following outcomes were included: (1) Lifetime prevalence (i.e., prevalence of subject reporting ever having a reaction or hypersensitivity to respective foods) and point prevalence (i.e., prevalence of subjects reporting having a reaction or hypersensitivity to respective foods currently or during the past 12 months) of self‐reported FA; (2) lifetime and point prevalence of self‐reported physician‐diagnosed FA (i.e., doctor‐diagnosed FA reported by a subject in a questionnaire); (3) point prevalence of specific immunoglobulin E (sIgE) sensitization; (4) point prevalence of skin prick test (SPT) sensitization; (5) point prevalence of symptoms plus sIgE sensitization; (6) point prevalence of symptoms plus SPT sensitization; (7) point prevalence of food challenge (oral food challenge [OFC] or double‐blind placebo‐controlled food challenge [DBPCFC]) positivity; and (8) point prevalence of food challenge positivity (OFC or DBPCFC) and/or clinical history of FA (i.e., FA confirmed by a convincing clinical judgment by a physician without food challenge).

### 2.3 Risk of bias assessment

The risk of bias assessment for individual studies was carried out by the same pairs of reviewers who completed the screening procedure by employing an adapted version of the Critical Appraisal Skills Programme (CASP; http://www.casp‐uk.net) quality assessment tool. Conflicts between the pairs were resolved by consensus or by consulting the project PI (BN).

### 2.4 Data analysis and synthesis

Data from each included study were collected using a customized data extraction form. When sufficient data were available, we recalculated the estimates using minimally measured events rather than extrapolated ones. Meta‐analysis was considered meaningful for all outcomes with three or more records available. To obtain the 95% confidence intervals (95% CI), we employed the Wilson score method without continuity correction.^7^ In case of the need of clarification regarding the data presented in a study, a request of clarification was sent to the corresponding author of the said paper. Following the revised protocol of the 2014 review, we attempted to stratify the available data by age groups (children [0–17 years], and adults [≥18 years]), and by European regions (Northern, Eastern, Southern, Western Europe) according to the classification by the United Nations (see Appendix 1). For this review, we analyzed the geographical distribution of the studies reporting on FA/FS only for foods that were reported in at least four studies (equaling the total number of European regions according to the United Nations classification, i.e., four regions).

We performed random‐effects meta‐analysis to derive pooled prevalence estimates for individual FAs from all studies that provided adequate numerical data and shared methodologically comparable data during 2012–2021. Because of the scarcity of data, it was not possible to perform meta‐analysis by age and by European region. We employed the Stata software (StataCorp. 2019, Stata Statistical Software: Release 16. StataCorp LLC) to complete the analysis and used the *I* ^2^ statistic to assess heterogeneity across studies. We included European countries within and without the Organization for Economic Cooperation and Development (OECD) in the meta‐analysis.

We further compared the pooled prevalence estimates obtained from random effect meta‐analysis for FA/FS to foods other than the eight big foods with our recently published 2012–2021 pooled prevalence estimates for FA/FS to the eight big foods.^4^

## 3 RESULTS

**FIGURE 1:**
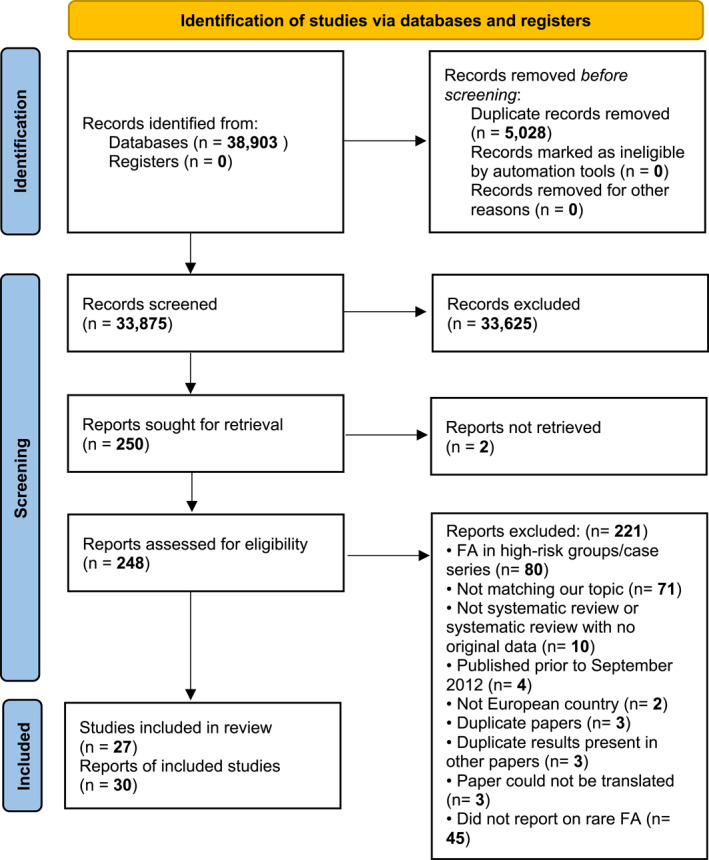
PRISMA flow diagram for systematic review on prevalence of food allergy outside the eight big FAs between September 2012 and June 2021.

The study selection and screening process is presented in the PRISMA flow chart (Figure 1). We identified 38,903 records published between 2012 and 2021. After the screening process, 27 studies reporting on FA/FS other than the eight big FAs were included.^7^, ^8^, ^9^, ^10^, ^11^, ^12^, ^13^, ^14^, ^15^, ^16^, ^17^, ^18^, ^19^, ^20^, ^21^, ^22^, ^23^, ^24^, ^25^, ^26^, ^27^, ^28^, ^29^, ^30^, ^31^, ^32^, ^33^, ^34^, ^35^, ^36^ Table 1 summarizes the main characteristics (i.e., age of the subjects involved, type of study, etc.) and the overall risk of bias score of the included studies. Of the 27 included studies, 18^9^, ^10^, ^11^, ^14^, ^15^, ^17^, ^20^, ^21^, ^22^, ^23^, ^24^, ^25^, ^26^, ^27^, ^28^, ^29^, ^30^, ^31^, ^32^, ^35^ were cross‐sectional and nine^12^, ^13^, ^16^, ^18^, ^19^, ^34^, ^36^, ^37^ were cohort studies. Three of the studies were international multi‐center studies, reporting multiple estimates on the same allergenic foods/outcome (one estimate from each country).^9^, ^10^, ^11^, ^14^, ^26^ Nineteen studies were undertaken only in children, five studies were undertaken only in adults, two studies reported estimates for both children and adults, while in one study the age of the participants included was not reported. Most studies were graded at a moderate risk of bias. The grading of the main features of the CASP quality assessment tool for each study is summarized in Figure S1.

**TABLE 1:** Main characteristics and overall risk of bias of the studies investigating the prevalence and incidence of FAs outside the eight big FAs in Europe between September 2012 and June 2021.

Reference, country	Study design	Study population	Age of subjects	Method of outcome assessment	Type of reported food allergy/sensitization	Overall risk of bias assessment
		*N* (children/adults)				
		number included				
Bröms et al. 2013, Sweden^8^	Cross‐sectional study	ODC: 4886 *n* = 1119	1–6 years old	Self‐reported	Stone fruits	Weak
		1–2 years old 1119				
		3–4 years old 1993				
		3–4 years old 1993				
Burney et al. 2014, Le et al., 2015, Lyons et al. 2019, Switzerland, Spain, The Netherlands, Poland, Bulgaria, Greece, Lithuania, Iceland^9^, ^10^, ^11^	Cross‐sectional study	All: 17,366	20–54 years old	Self‐reported, sIgE, DBPCFC	Peach, apple, celery, carrot, kiwi, tomato, sesame, banana, corn, sunflower, poppy, melon, buckwheat, lentils, mustard	Moderate
		Switzerland 2250				
		Spain 943				
		The Netherlands 3865				
		Poland 1499				
		Bulgaria 2118				
		Greece 1979				
		Lithuania 2598				
		Iceland 2114				
Clausen et al. 2017, Sweden^12^	Cohort study	3637	12 years old	SR physician diagnosis + clinical history	Peas, stone fruits	Moderate
De Jong et al. 2019, The Netherlands^13^	Cohort study	5471 (*n* = 4078 included subjects for peach allergy prevalence computation)	10 years old	SPT	Peach	Moderate
Dereci et al. 2015, Haktanir et al. 2017, Turkey^14^, ^15^	Cross‐sectional study	15,783	6–18 years old	Self‐reported, SPT, PTP, OFC, DBPCFC	Blueberry and kiwi allergy	Strong
Doğruel et al. 2016, Turkey^16^	Cohort study	1377 (but only 920 where sIgE and SPT tested)	0–12 months	SPT and/or s‐IgE, SPT, s‐IgE, OFC	Chicken, beef, banana	Moderate
Gaspar‐Marques et al. 2014, Portugal^17^	Cross‐sectional study	1217	0–6 years	Self‐reported	Strawberry, chocolate, kiwi, orange, peach	Moderate
Grabenhenrich et al. 2020, Iceland, United Kingdom, the Netherlands, Germany, Poland, Lithuania, Spain and Greece^18^	Cohort study	All 6069	6–10 years old	Self‐reported, self‐reported physician diagnosis	Tomato, kiwi, strawberry, apple, citrus fruit	Moderate
		Iceland 945				
		United Kingdom 454				
		The Netherlands 652				
		Germany 1001				
		Poland 819				
		Lithuania 949				
		Spain 688				
		Greece 561				
Grimshaw et al. 2016, United Kingdom^19^	Cohort study	823	2 years old	DBPCFC or positive clinical history	Lentil, broccoli	Moderate
Haftenberger et al., Germany^20^	Cross‐sectional study	7025	18–79	sIgE	Chicken protein, flour, barley, rice, sesame, almond, tomato, carrot, potato, strawberry, apple, kiwi, celery, cherries, lupin seed, peach	Strong
Hicke‐Roberts et al. 2020, Sweden^21^	Cross‐sectional study	1027 (for cereal 995)	7–8 years old	Self‐reported	Cereals	Moderate
Ivakhnenko et al. 2013, Ukraine^22^	Cross‐sectional study	1000	0–30 months old	Self‐reported	Citrus, fruit, vegetables, chocolate	Moderate
Järvenpää et al. 2014, Finland^23^	Cross‐sectional study	1653	6–7 years old	Self‐reported	Legumes, spices, fruit and vegetables	Moderate
Jurado‐Escobar et al. 2017, Spain^24^	Cross‐sectional	1396	1–90 years old	SPT	Peach, any vegetable	Moderate
Kaya et al. 2013, Turkey^25^	Cross‐sectional study	10,096	11–15 years old	Self‐reported, sIgE, SPT, OFC, DBPCFC	Chocolate, tomato, kiwi, honey, sesame, black pepper, strawberry, honey, banana	Moderate
Lyons et al. 2020, Switzerland, Spain, Greece, Poland, Bulgaria, Lithuania, Iceland, and The Netherlands^26^	Cross‐sectional study	16,935	7–10 years old	Self‐reported, sIgE, DBPCFC	Peach, banana, tomato, celery, carrot, sesame seeds, apple, kiwi, melon, buckwheat, sunflower seed, poppy seed, corn, lentils mustard seeds	Strong
Mortz, et al. 2013, Denmark^27^	Cross‐sectional study	460	Age not indicated	sIgE, sIgE	Sesame	Moderate
Mustafayev et al. 2013, Turkey^28^	Cross‐sectional study	6963 (*n* = 5609 for OFC‐confirmed allergy to specific foods)	10–11 years old	OFC	Beef meat, peach, spinach, cheese, kiwi	Moderate
Patelis et al. 2014, Sweden and Iceland^29^	Cross‐sectional study	2307	20–54 years old	Self‐reported	Fruit, vegetables, chocolate, meat, herbs, chilli, and garlic	Moderate
Raciborski et al. 2012, Poland^30^	Cross‐sectional study	1801	6–8 years old	Self‐reported	Chocolate, and fruit	Moderate
Rentzos 2019 et al., Sweden^31^	Cross‐sectional study	18,083	16–75 years old	Self‐reported	Anise/caraway, apricot, apple, avocado, banana, bean, beef, camomile, carrot, cayenne/red pepper, cheese, chicken, celery, cherry, chilli/tabasco, chocolate, coriander, dried fruit, flour (non wheat), fried/fat food, kiwi, lingonberry, melon, nectarine, orange, parsley, pea, peach, pear, plum, poppy seed, potato, pork, red meet, salami, sesame, strawberry, sunflower, sweet potato, tomato	Moderate
Skypala et al. 2013, United Kingdom^32^	Cross‐sectional study	3590	18–75 years old	Self‐reported	Non‐citrus fruit, vegetables, food additives, citrus fruit, curry and spices, tomato, seeds, beans and lentils	
Stefanaki et al. 2018, Greece^33^	Cohort study	917	4 years old	Self ‐reported	Tomato	Moderate
Strinnholm et al. 2014, Sweden^34^	Cohort study	2585	7–8 years old	Self‐reported	Fruit and nuts, kiwi, orange, apple, carrot, banana	Moderate
Topcu et al. 2019, Turkey^35^	Cross‐sectional	4932	6–7 years old	Self‐reported, SPT, sIgE, DBPCFC	Beef	Moderate
Venkataraman et al. 2017, United Kingdom^36^	Cohort study	1 year old 1268	0–18 years old	Self‐reported	Fruit, tomato, kiwi	Moderate
		2‐year‐old 1159				
		4‐year‐old 1217				
		10 years old 1368				
		18‐year‐old 1290				
Westerlaken‐van Ginkel et al. 2020, The Netherlands^37^	Cohort study	78,890	Adult	Self‐reported	Apple, kiwi, sesame, strawberry, cherry, pear, peach, banana	Moderate

*Note*: Data were extracted from conference abstracts or poster in the following studies: Dereci et al. 2015; Jurado‐Escobar et al. 2017; Stefanaki et al. 2018; Raciborski et al. 2012; Mortz et al. 2013; Topcu et al. 2019. For Clausen et al. 2017, data were extracted from a university thesis. The following papers/abstracts included in Table 1 reported about the same study population: Dereci et al. 2015, together with Haktanir et al. 2017; Lyons et al. 2019, together with Burney et al. 2014, and Le et al. 2015.

Abbreviations: DBPCFC, double‐blinded placebo‐controlled food challenge; FA, food allergy; FS, food sensitization; OFC, oral/open food challenge; PTP, prick to prick skin test for sensitization to specific foods; sIgE, specific immunoglobulin E test; SPT, skin prick test for sensitization to specific foods; SR, self‐reported FA.

### 3.1 Frequency of FA

The detailed results on the prevalence and incidence of the FAs are summarized in Tables S1 and S2.

Table 2 summarizes the ranges of prevalence and incidence for all the FAs identified in this review using different assessment methods (i.e., point prevalence self‐reported FA, point prevalence sIgE sensitization). For synthesis purposes, we have divided the different foods into the following five categories: fruits, vegetables/legumes, herbs/seeds/condiments/spices, cereals, meat, and other foods.

**TABLE 2:** Summary of range of estimates of lifetime and point prevalence of individual FAs outside the eight big FAs in Europe by different methods of assessment from studies published between September 2012 and June 2021.

Type of food allergy	Number of studies	Lifetime prevalence (%)		Point prevalence and cumulative incidence^a^ (%)						
		SR	SR physician‐ diagnosed	SR	SR physician‐ diagnosed	sIgE positivity	Symptoms + sIgE positivity	SPT positivity	Clinical history or FC (OFC or DBPCFC) positivity	FC (OFC or DBPCFC) positivity
Fruit										
Fruits	4	9.32		0.29–8.01						
Apple	7	0.11–0.98	0.01–0.73	2.35–8.40		2.05–11.95	0–1.90			0.05–0.13
Apricot	1			1.7						
Avocado	1			0.85						
Banana	7			0.14–1.35		0.01–15.47	0–0.95	0.01		0.01
						0^a^		0.11^a^		0.07^a^
Blueberry	1	0.00051						0.02		0.0000634
Cherries	3			0.22–3.1		10.1				
Citrus fruits	3	0.01–18.10	0.01–0.73							
Kiwi	12	0.01–1.02	0.01–0.58	0.23–8.05		0.01–9.53	0–1.35	0.01–0.11		0.01–0.10
Lingonberry	1			0.1						
Melon	3			0.3–0.4		0.81–9.19	0–0.95			
Nectarine	1			2.4						
Non‐citrus fruit	1	4.7								
Orange	3			0.49–4.53						
Peach	9			0.20–3.25		2.49–13.21	0–2.02	2.1–3.8		0.02–0.05
Pear	2			0.12–4.0						
Plum	1			3.0						
Strawberry	6	0.01–1.1	0.01–0.49	0.30–2.70		0.01–5.5				
Stone fruits	2			0.8–1.35	1.62					
Tomato	9	0.01–1.76	0.01–0.49	0.16–2.1		2.55–13.27	0–0.63			0.01
Vegetables and legumes										
Vegetables	3	3.3–4.7								
Bean	1			1.8						
Broccoli	1								0.1^a^	
Carrot	5			0.45–3.15		2.11–12.46	0–1.01			
Celery	4			0.3		2.42–13.09	0–1.24			
Lentils	4					2.11–7.99	0–0.56		0.1^a^	
Legumes	1			0.8						
Lupin seed	1					3.9				
Peas	2			0.7	0.3					
Potato	2			1.6		5.05				
Sweet pepper	1			2.1						
Red pepper	1			1.6						
Spinach	1									0.02
Herbs, condiments, seeds and spices										
Anise/caraway	1			0.2						
Black pepper	1					0.01				
Buckwheat	2					1.37–8.89	0–0.14			
Chili/tabasco	1			2.2						
Coriander	1			0.1						
Herbs, chilli, garlic	1	0.87								
Mustard seed	2					0.37–4.92	0–0			
Parsley	1			0.3						
Poppy seeds	3			0.1		0.75–8.50	0–0			
Seed	1	0.7								
Sesame seeds	7			0.1–0.15		1.29–12.10	0–0.15	0.01–3.91		0.01
Spices	2	1.3		0.5						
Sunflower seeds	3			0.1		1.37–8.89	0–0.53			
Cereals										
Cereals	1			0.7^a^						
Barley	1					6.3				
Corn	2					1.80–9.43	0–0.18			
Flour (non wheat)	1			0.7		6.0				
Rice	1					4.0				
Rye flour	1					6.0				
Meat										
Beef	4			0.4–2.60		0.22^a^		0.11^a^		0.04–0.30
Chicken	3			0.1		1.6		0.22^a^		0.41^a^
						0.11^a^				
Meat	1	0.82								
Pork	1			0.6						
Red meat	1			0.9						
Salami	1			0.5						
Other food allergies										
Camomile	1			0.6						
Cheese	2			0.8						0.02
Chocolate	6	1–12.3		1.4–10.1				0.01		0.01
Dried fruit	1			0.3						
Food additives	1	1.61								
Fried/fat food	1			3.7						
Honey	1					0.01		0.01		0.01

Abbreviations: DBPCFC, double‐blind, placebo‐controlled food challenge; FC, food challenge; OFC, oral/open food challenge; PTP, prick by prick skin test for sensitization to specific foods; sIgE, specific immunoglobulin E test; SPT, skin prick test for sensitization to specific foods; SR, self‐reported FA.

^a^ Cumulative incide.

The total number of reported FAs outside the eight big FAs was 66. Kiwi was the most studied food (reported in 12 studies), followed by peach and tomatoes (9 studies each), apple, banana, and sesame seed (7), strawberry and chocolate (6), carrots (5), celery, lentils, and beef (4). Thirty‐seven of the 66 foods were reported in only one study, among them being various types of meat (e.g., red meat, pork, salami), specific types of fruits (e.g., nectarine, plum), vegetables (e.g., broccoli), as well as different types of condiments (e.g., chili). Twelve foods were reported in at least four studies, that is, kiwi, peach, tomato, sesame seed, apple, banana, strawberry, chocolate, carrot, celery, lentils, and beef. Figure S2 presents the distribution by the European region of the studies reporting on these 12 FA/FS.

**FIGURE 2:**
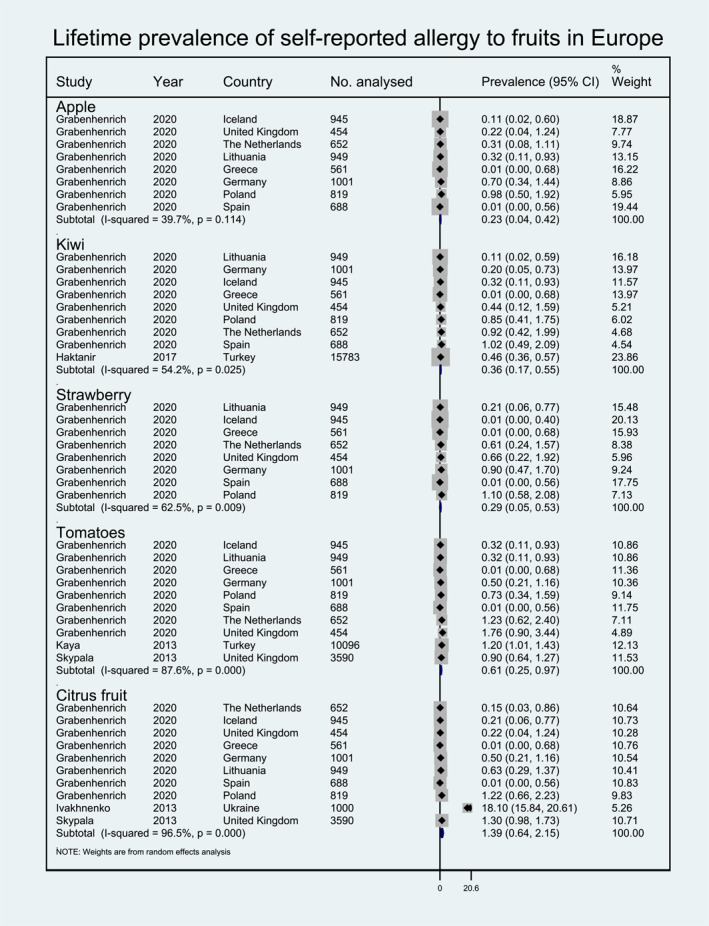
Lifetime prevalence of self‐reported allergy to fruits in Europe.

**FIGURE 3:**
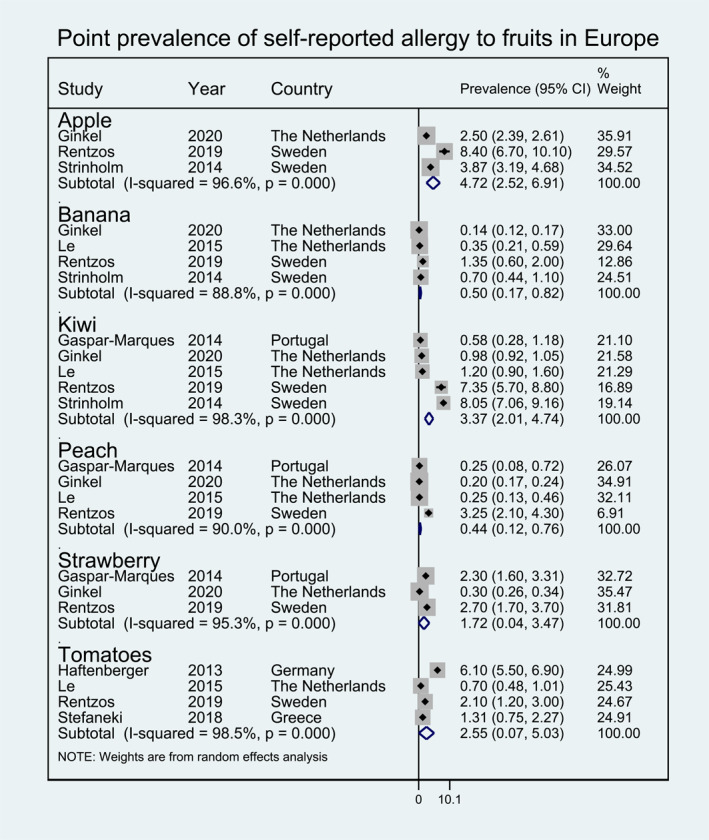
Point prevalence of self‐reported allergy to fruits in Europe.

**FIGURE 4:**
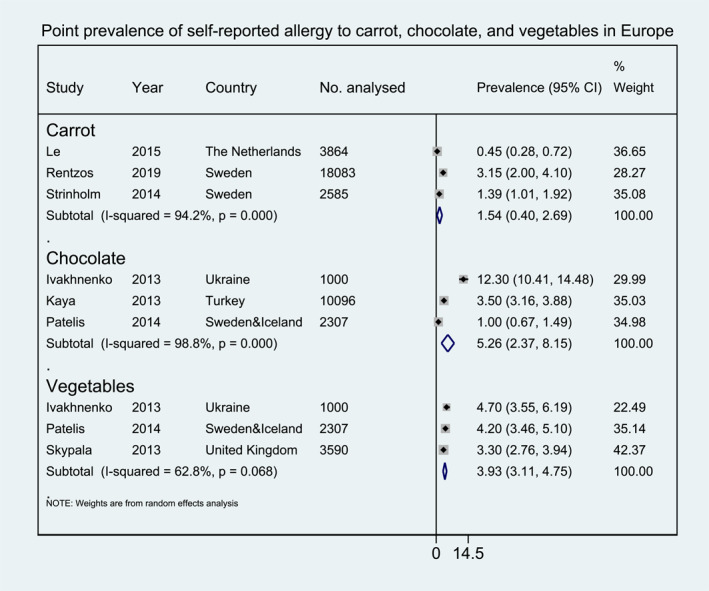
Point prevalence of self‐reported allergy to carrots, chocolate, and vegetables in Europe.

**FIGURE 5:**
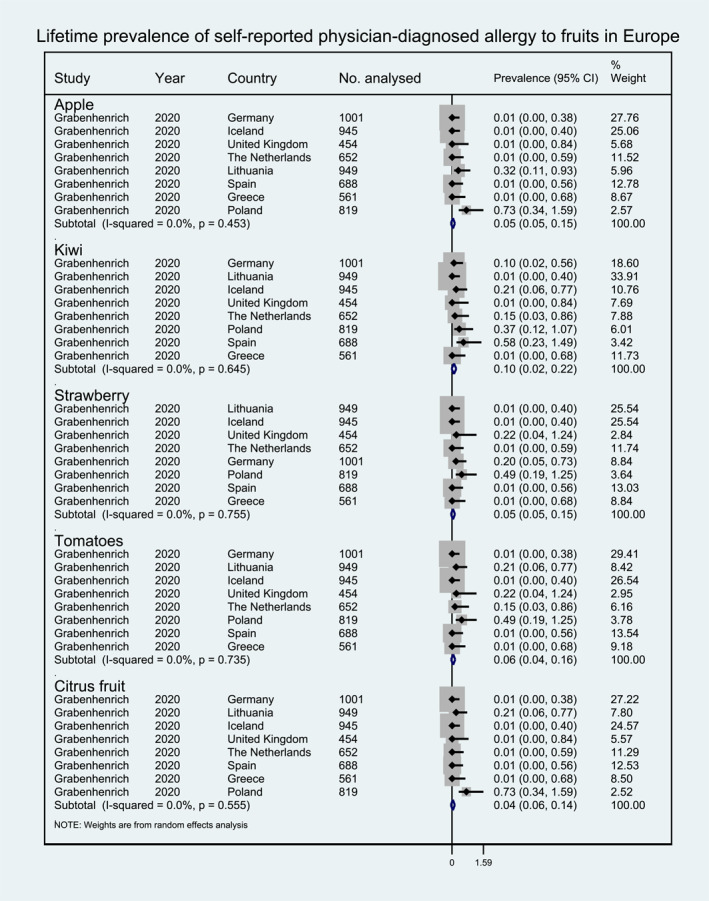
Lifetime prevalence of self‐reported physician‐diagnosed allergy to fruits in Europe.

**FIGURE 6:**
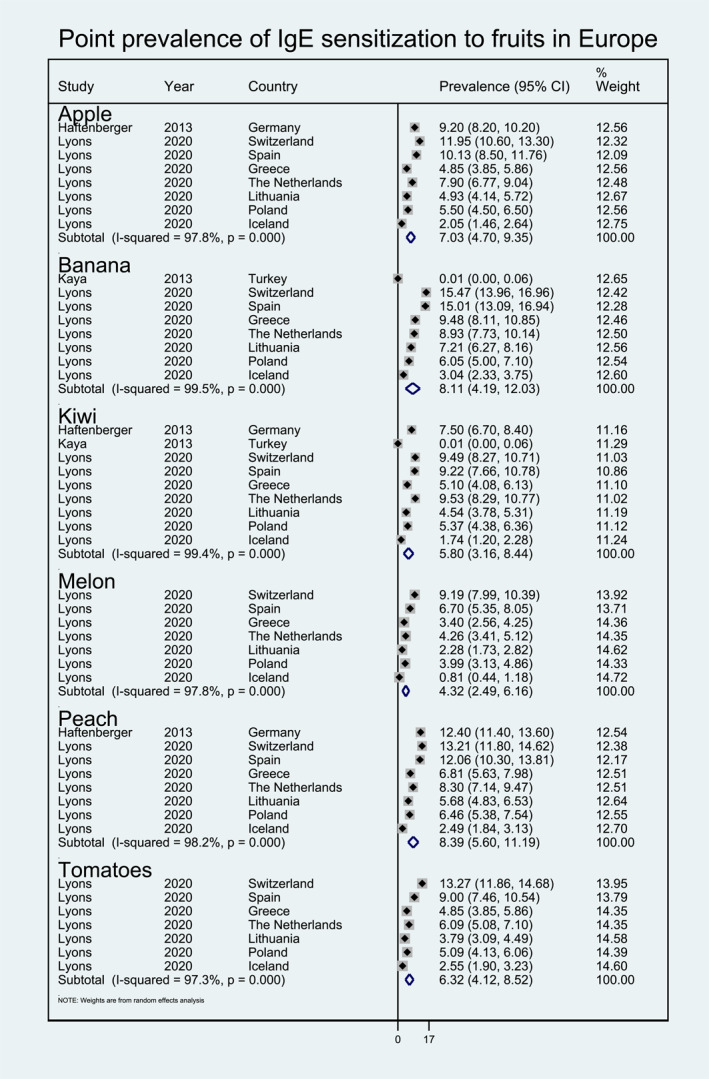
Point prevalence of immunoglobulin E sensitization to fruits in Europe.

**FIGURE 7:**
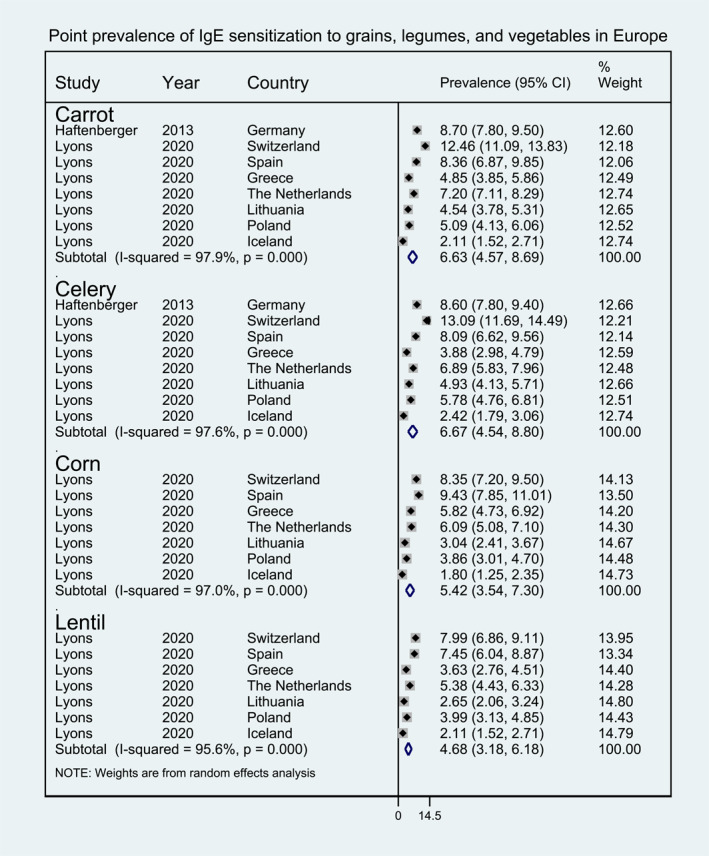
Point prevalence of immunoglobulin E sensitization to grains, legumes, and vegetables in Europe.

**FIGURE 8:**
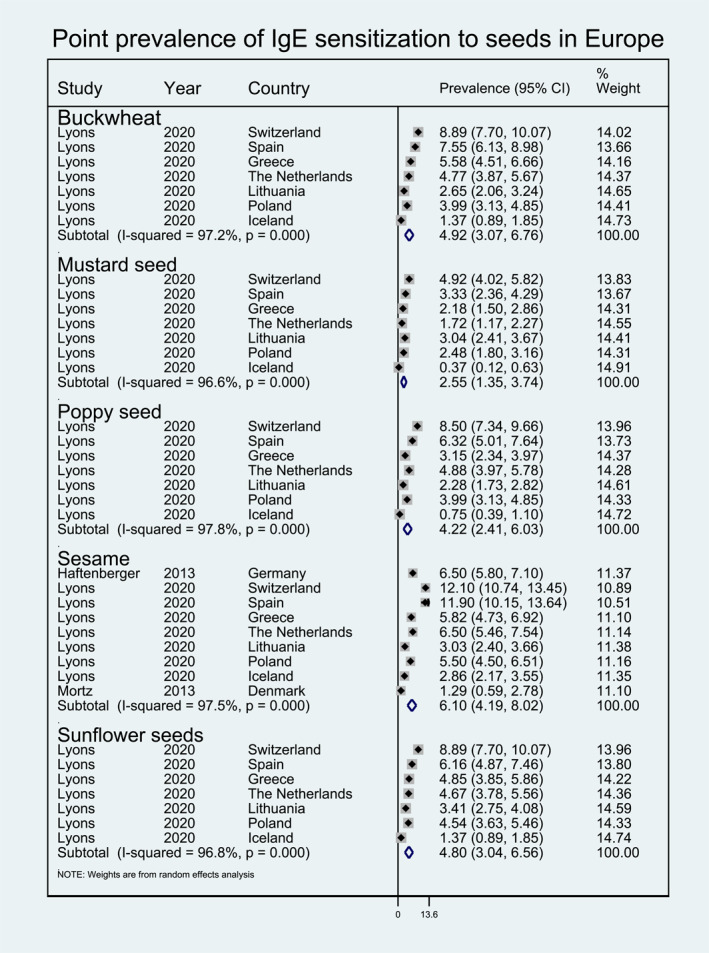
Point prevalence of immunoglobulin E sensitization to seeds in Europe.

Meta‐analysis was performed for 19 foods for which enough data were available to allow pooling of results for at least one of the outcomes investigated (i.e., point prevalence self‐reported FA, FC‐verified FA etc.). The pooled lifetime and point prevalence estimates for each FA/FS according to the different outcomes investigated are presented in Figures 2, 3, 4, 5, 6, 7, 8. There was significant heterogeneity among the studies pooled in the meta‐analysis (*I* ^2^ ≥ 80 in each case). Due to the paucity of data, we could not observe any consistent pattern across age groups.

### 3.2 Self‐reported food allergy

In total, 19 studies reported on self‐reported FA, one of which was an international multi‐center study reporting multiple estimates for each FA/outcomes investigated (one from each center/country). The highest prevalence of self‐reported FA was 18.1% for the lifetime prevalence of citrus fruit and 10.1% for the point prevalence of chocolate, respectively. The lowest lifetime prevalence of self‐reported FA was reported for blueberry (0.0005%). The lowest point prevalence was reported in chicken, coriander, lingonberry, poppy and sesame seed, and sunflower allergy (0.1%).

Pooled self‐reported lifetime prevalence was available only for fruits, including estimates for citrus fruit (1.39%, 95% CI 0.64, 2.15), tomato (0.61%, 95% CI 0.25, 0.97), kiwi (0.36%, 95% CI 0.17, 0.55), strawberry (0.29, 95% CI 0.05, 0.53), and apple (0.23%, 95% CI 0.04, 0.42) (Figure 2).

Pooled self‐reported point prevalence of allergy to fruit was available for apple (4.72%, 95% CI 2.52, 6.91), kiwi (3.37%, 95% CI 2.01, 4.74), tomato (2.55%, 95% CI 0.07, 5.03), strawberry (1.72%, 95% CI 0.04, 3.47), banana (0.50%, 95% CI 0.17, 0.82), and peach (0.44%, 95% CI 0.12, 0.76) (Figure 3). Pooled self‐reported point prevalence of allergy to vegetables was available only for any vegetable (3.93%, 95% CI 3.11, 4.75) and carrot (1.54%, 95% CI 0.40, 2.69) (Figure 4). Finally, pooled self‐reported point prevalence of allergy to chocolate was 5.26% (95% CI 2.37, 8.15) (Figure 4).

### 3.3 Self‐reported physician‐diagnosed food allergy

Lifetime prevalence and point prevalence of self‐reported physician‐diagnosed FA were investigated in one study. The study reporting on lifetime prevalence was an international multi‐center study and provided sufficient data to perform meta‐analysis.^17^ The highest estimate for lifetime prevalence was 0.7% for allergy to citrus fruit and apple, while the lowest estimate was reported for the kiwi allergy (0.1%). The study reporting on point prevalence only reported prevalence estimates for stone fruits (1.6%), and for peas (0.3%).^11^ Pooled lifetime prevalence estimates for self‐reported physician‐diagnosed FAs were only available for fruits: tomato (0.06%, 95% CI 0.04, 0.16), apple (0.05%, 95% CI 0.05, 0.15), strawberry (0.05%, 95% CI 0.05, 0.15), citrus fruit (0.04%, 95% CI 0.06, 0.14), and kiwi (0.10%, 95% CI 0.02, 0.22) (Figure 5).

### 3.4 SPT or sIgE sensitization

Ten studies reported FA by means of sensitization (positive SPT or sIgE test) to foods, and two were international multi‐center studies. Six studies reported estimates for sIgE positivity, while seven reported estimates for SPT positivity. For positivity in sIgE tests, the FA with the highest reported point prevalence estimates was banana (15.4%), while the lowest prevalent were kiwi, black pepper, strawberry, and honey (each at 0.01%).

For SPT positivity, the highest estimate was reported for sesame allergy (3.9%), followed by peach allergy (3.8%). Kiwi, chocolate, honey, banana, and sesame had the lowest point prevalence estimates for SPT‐positive sensitization (each at 0.01%). Data on SPT positivity were too scarce to perform a meta‐analysis.

Within fruits, pooled point prevalence of sIgE sensitization was available for peach (8.39%, 95% CI 5.60, 11.19), banana (8.11%, 95% CI 4.19, 12.03), apple (7.03%, 95% CI 4.70, 9.35), tomato (6.32%, 95% CI 4.12, 8.52), kiwi (5.80%, 95% CI 3.16, 8.44), and melon (4.32%, 95% CI 2.49, 6.16) (Figure 6). Pooled point prevalence of sIgE sensitization was only available for carrot (6.63%, 95% CI 4.57, 8.69), celery (6.67%, 95% CI 4.54, 8.80), and lentils (4.68%, 95% CI 3.18, 6.18) within the vegetable/legumes category, and for corn (5.42%, 95% CI 3.54, 7.30) within the cereal category (Figure 7). Finally pooled point prevalence sIgE sensitization was also available for various types of seeds, including sesame seed (6.10%, 95% CI 4.19, 8.02), buckwheat (4.92%, 95% CI 3.07, 6.76), sunflower seed (4.80%, 95% CI 3.04, 6.56), poppy seed (4.22%, 95% CI 2.41, 6.03), mustard seed (2.55%, 95% CI 1.35, 3.74) (Figure 8).

### 3.5 Challenge‐verified food allergy

From the 10 studies that performed food challenges (OFC or DBPCFC), the highest prevalence estimate was for beef allergy (0.3%), while the lowest was negligibly for blueberry allergy.

Data on FC sensitization were too scarce to perform meta‐analysis.

### 3.6 Comparison of pooled prevalence estimates for food allergy or sensitization outside the eight big foods and pooled estimates for the eight big food allergies

Box S1 in the Supporting Information S1 presents the comparison between the pooled prevalence estimates obtained for FA/FS outside the eight big foods and our recently published pooled prevalence estimates for the eight big FA/FS.^4^ We have compared estimates for lifetime prevalence of self‐reported FA and of self‐reported physician‐diagnosed FA, as well as for point prevalence of self‐reported FA and of sIgE‐positive FA/FS. Lifetime prevalence of self‐reported physician‐diagnosed FA was higher for the commonly studied cow's milk, egg, fish, peanut, tree nuts, wheat, soy, and shellfish FA/FS (i.e., the eight big FA/FS) compared to other foods. For all the other outcomes (i.e., lifetime prevalence of self‐reported FA, point prevalence of self‐reported FA and of sIgE positive FA/FS), we did not observe a clear pattern in terms of highest/lowest prevalence between FA/FS and the eight big foods and FA/FS to other foods.

## 4 DISCUSSION

### 4.1 Statement of principal findings

For the first time, this systematic review provides a comprehensive view on the frequency of FA/FS outside the eight so‐called big FAs (i.e., cow's milk, egg, wheat, soy, peanut, tree nuts, fish, and shellfish allergy). The most frequently investigated food was kiwi. The overall pooled estimates for kiwi allergy or sensitization was 0.4%, 3.4%, and 5.8% for self‐reported lifetime, self‐reported point, and point prevalence of sIgE sensitization, respectively. Among vegetables, carrot allergy or sensitization was the most reported, with pooled prevalence of 1.5% and 6.6% for point prevalence self‐reported allergy and sIgE sensitization, respectively. Beyond fruits and vegetables, other frequently investigated foods were sesame seeds (7 studies), chocolate (6), lentils (4), and beef (4). Sesame seed, which is included among the foods that need to be labeled on pre‐packed foods when used as ingredients under the EU 1169/2011 regulation, gave a pooled prevalence of 6.1% for sIgE sensitization. Most other FAs/FS were very rarely reported, 37 only in one study, which limited our ability to derive a clear picture of their prevalence in Europe. Given the paucity of data, no consistent patterns in prevalence could be seen by European region and between children and adults across the FAs investigated.

Finally, most studies were rated as having a “moderate” risk of bias, indicating that the methodology through which evidence‐based data on FAs are derived needs to be improved, in order to gain a better appreciation of the frequency of FA across Europe.

### 4.2 Strengths and limitations of the current update

This systematic review and meta‐analysis was conducted following a rigorous methodology and a systematic approach in every phase of the study. We completed a comprehensive literature search of six major electronic databases, including two databases which were not previously considered in the 2014 review. Moreover, compared to the 2014 review, we included more keywords in the database search to limit the possibility of missing any relevant information. Including new databases and keywords allowed us to take into consideration the advances that have been made since the publication of the 2014 systematic review.

We included all methods of assessment of FA or FS and did not apply any language restriction during database searches and literature screening, which allowed us to provide a comprehensive picture of the frequency of allergies to foods beyond the eight big FAs in Europe.

Conversely, the quality of our study is partly constrained by the limited number of data available for some of the allergenic foods investigated, including some foods that are commonly perceived as important allergenic foods. For instance, although lupins are listed among the 14 food ingredients that need to be labeled under the EU 1169/2011 regulation, only one study reported on the prevalence of lupin allergy/sensitization by measuring sIgE positivity.^20^ Similarly, mustard seed allergy/sensitization, which is also included among the food ingredients regulated by the EU 1169/2011, was investigated only by means of sIgE positivity and by sIgE positivity plus allergic symptoms in two studies. However, both studies were part of a large European initiative called the EuroPrevall project, which investigated FA frequency in different European countries; therefore, data on the prevalence of mustard sensitization were available from different centers/countries.^9^, ^10^, ^11^, ^26^

As already observed in the systematic review published on the prevalence of any FA/FA to the eight big foods, the high prevalence observed for self‐reported FA and for sIgE/SPT FS for some of the foods investigated did not match the low prevalence estimates obtained by measuring FC‐verified allergy. However, even though FC is considered the gold standard methodology to assess FA, its use was very restricted in the studies included in the review. The correct assessment of FA would be improved through increased use of the gold standard FC measurement.

The high heterogeneity observed across studies may be a sign of methodological discrepancy in the way FA/FS was addressed by the authors of the included studies (e.g., different methods of assessment for the considered outcomes), but it could also indicate that there is a wide variation in the prevalence of FA/FS within and between ages and European regions. Considering these two possible interpretations for the encountered high heterogeneity, the data resulting from the analysis of the studies should be evaluated with caution.

One of the limitations of the review is that most of the selected studies did not differentiate between IgE and non‐IgE allergy, which prevented us from distinguishing between IgE‐mediated and non‐IgE‐mediated FA phenotypes.

Finally, albeit illogical, pooled estimates for lifetime prevalence of self‐reported FA to apple, kiwi, strawberries, and tomato were lower than point prevalence. This inconsistency can however be explained by the fact that for most of the foods investigated, lifetime and point prevalence were not pooled from the same studies.

### 4.3 Comparison of findings to previous studies on allergy or sensitization to foods other than the eight big foods

To our knowledge, this is the first systematic summary of the prevalence of allergy or sensitization to foods other than the eight so‐called big foods. Before our study, Zuidmeer et al. (2008) published a systematic review of the worldwide prevalence of plant FA, defining six plant food categories, that is, fruits, vegetables/legumes, tree nuts, soy, wheat, and sesame seed/cereals/spices/herbs.^38^ For all the other food types within the categories investigated by the authors, only one estimate was available. Notably, although the authors investigated plant FA worldwide, most of the estimates available came from European studies. Another interesting observation is that, although Zuidmeer et al. investigated four FA outcome, that is perceived FA, sIgE‐positive FS, SPT‐positive FS, and FC‐verified FA, for the fruit, vegetables/legumes and sesame seed/cereals/spices/herb category, there were no available data on sIgE sensitization, while more data were available on SPT sensitization. This result is almost opposed to what was observed in our review where sIgE sensitization was far more represented than SPT, which may suggest that in vitro food specific IgE testing is now preferred over SPT, although the two methodologies have different sensitivity and specificity and do not always concord.

### 4.4 Comparison of findings to previous findings on allergy or sensitization to the eight big foods

Although cow's milk, egg, wheat, soy, peanut, tree nuts, fish, and shellfish are traditionally considered the eight most common allergenic foods in Europe, pooled prevalence estimates for the eight big FA/FS were not always the highest compared to FA/FS to other foods in Europe between 2012 and 2021. Indeed, especially for point prevalence of self‐reported FA, and for point prevalence of sIgE‐positive FA/FS, some of the foods investigated in this review were found to have a higher prevalence than many of the eight big foods.

However, the number of available prevalence estimates for foods other than the eight big foods was frequently lower than the available estimates for allergy or sensitization to the eight big foods, which can result in a less accurate meta‐analysis.

### 4.5 Interpretation and implication of the current findings

As already mentioned, the results of the systematic review and meta‐analysis should be interpreted with caution. The fact that most of the studies were graded as at moderate risk of bias according to the CASP quality assessment tool, combined with the high heterogeneity observed across studies, prevents the derive of univocal conclusions on the study findings.

More studies would be required to obtain a comprehensive view of the burden of FA outside the eight big FAs. Moreover, similar to what was observed in the updated review of any FA and common FA, future studies would benefit from the definition of shared protocols and standardized methodologies for the assessment of FA in Europe.

## 5 CONCLUSIONS

The data presented in this review suggest that allergy to some foods traditionally not considered important are now emerging as relevant FAs, including but not limited to kiwi, peach, tomato, sesame seed, apple, banana, strawberry, chocolate, carrot, celery, lentils, and meat. This observation may partly explain our recent finding that, although the prevalence of “any FA/FS” has increased in the last 10 years, the prevalence of FA/FS in cow's milk, egg, wheat, soy, peanut, tree nuts, fish and shellfish has not considerably changed over the same period. The focus on FA in Europe should therefore not be limited to the eight so‐called big FA, but rather extended to other types of foods which need to be considered both for clinical purposes and population risk assessment, including the labeling legislation, to improve the understanding of FA in Europe.

## AUTHOR CONTRIBUTIONS

Bright I. Nwaru and Graham Roberts defined the research question and the search strategies with assistance from Daniil Lisik. Daniil Lisik with the assistance from Bright I. Nwaru developed the data extraction form. Screening, data extraction, narrative synthesis was done by Giulia C. I. Spolidoro, Mohamed Mustafa Ali, Sungkutu Nyassi, Yohannes Tesfaye Amera, and Bright I. Nwaru. Manuscript writing was done by Bright I. Nwaru and Giulia C. I. Spolidoro. Antonella Muraro, Aziz Sheikh, Berber Vlieg‐Boerstra, Carina Venter, Ekaterina Khaleva, Graciela Rovner, Graham Roberts, Margitta Worm, and Ronald van Ree were consulted concerning methodology and synthesis of the findings. All authors (Athina Ioannidou, Antonella Muraro, Aziz Sheikh, Bright I. Nwaru, Berber Vlieg‐Boerstra, Carina Venter, Daniil Lisik, Ekaterina Khaleva, Graciela Rovner, Graham Roberts, Giulia C. I. Spolidoro, Mohamed Mustafa Ali, Margitta Worm, Ronald van Ree, Sungkutu Nyassi, Yohannes Tesfaye Amera) critically commented on drafts of the manuscript.

## CONFLICT OF INTEREST STATEMENT

Carina Venter reports grants (Reckitt Benckiser, Food Allergy Research and Education, and National Peanut Board) and personal fees (Reckitt Benckiser, Nestle Nutrition Institute, Danone, Abbott Nutrition, Else Nutrition, Sifter, and Before Brands). Ronald van Ree reports consultancies (HAL Allergy BV, Citeq BV, Angany Inc., Reacta Healthcare Ltd., Mission MightyMe, AB Enzymes, The Protein Brewery, and Unilever India), speaker's fees (HAL Allergy BV, ThermoFisher Scientific, and ALK), and stock options (Angany Inc.). Margitta Worm reports grants and personal fees (Stallergènes, HAL Allergy, Bencard Allergie, Allergopharma, ALK‐Abello, Mylan Germany, Actelion Pharmaceuticals Deutschland, Biotest, AbbVie Deutschland, Lilly Deutschland Aimmune, DBV Technologies SA, Regeneron Pharmaceuticals, Sanofi Aventis, Leo Pharma, Novartis, and Viatris) outside the submitted work and being past WAO co‐chair of the anaphylaxis committee and past chair of the food allergy interest group of EAACI. Berber Vlieg‐Boerstra reports personal and speaker fees (Vinimini, Nestlé, and Nutricia) and grants (Nutricia). Antonella Muraro reports grants and speaker's fees (Aimmune), speaker's fees (DVB Technologies SA, Viatris [Mylan], ALK, and Nestlé), and being a member of the Executive Committee of GA2LEN and past president of EAACI. Graham Roberts reports grants (Asthma UK and National Institutes of Health Research). Bright Nwaru reports unrestricted grants and personal fees from DBV Technologies and AstraZeneca, respectively. Giulia C.I. Spolidoro, Yohannes Tesfaye Amera, Mohamed Mustafa Ali, Sungkutu Nyassi, Daniil Lisik, and Athina Ioannidou report fees from ACT Institutet Sweden. The other authors report no conflicting interests related to this work. The funder played no role in the content and decision to submit this manuscript.

## Supporting information

Supporting Information S1

## Data Availability

The data that support the findings of this study are available from the corresponding author upon reasonable request.

## APPENDIX 1 GEOSCHEME OF EUROPEAN COUNTRIES BY UN

**Table:**

Eastern Europe	Northern Europe	Southern Europe	Western Europe
Belarus	Åland*	Albania	Austria
Bulgaria	Channel Islands (Guernsey, Jersey, Sark)	Andorra	Belgium
Czech Republic	Denmark	Bosnia and Herzegovina	France
Hungary	Estonia	Croatia	Germany
Poland	Faroe Islands	Gibraltar	Liechtenstein
Moldova	Finland	Greece	Luxembourg
Romania	Iceland	Holy See (Vatican City)	Monaco
Russia	Ireland	Italy	Netherlands
Slovakia	Isle of Man	Kosovo*	Switzerland
Ukraine	Latvia	Malta	
	Lithuania	Montenegro	
	Norway	(North) Macedonia	
	Svalbard and Jan Mayen Islands*	Portugal	
	Sweden	San Marino	
	UK (England, Scotland, Wales, and Northern Ireland)	Serbia	
		Slovenia	
		Spain	
		Turkey*	
		Yugoslavia (historical)*	
