# Supplementary material for: Food allergy outside the eight big foods in Europe: A systematic review and meta‐analysis
Source: Clin Transl Allergy. 2024 Feb 7;14(2):e12338. doi: 10.1002/clt2.12338 (PMC10848175; doi:10.1002/clt2.12338)
Supplement: Supplementary file 1 — Supporting Information S1 [file CLT2-14-e12338-s001.docx]

**Food allergy outside the eight big foods in Europe: a systematic review and meta-analysis**

Short title: **Food allergies outside the eight big food allergies in Europe**

Giulia C.I. Spolidoro^1^, Sungkutu Nyassi^2^, Daniil Lisik^2^, Athina Ioannidou^2^, Mohamed Mustafa Ali^3^, Yohannes Tesfaye Amera^3^, Graciela Rovner^4, 5^, Ekaterina Khaleva^6^, Carina Venter^7^, Ronald van Ree^8^, Margitta Worm^9^, Berber Vlieg-Boerstra^10, 11^, Aziz Sheikh^12^, Antonella Muraro^13^, Graham Roberts^6, 14^, Bright I. Nwaru^2, 15^

^1^Department of Clinical Sciences and Community Health, University of Milan, Milan, Italy

^2^Krefting Research Centre, University of Gothenburg, Gothenburg, Sweden

^3^School of Public Health and Community Medicine, Institute of Medicine, University of Gothenburg, Gothenburg, Sweden

^4^ACT Institutet Sweden, Vejbystrand, Sweden

^5^Division of Physiotherapy, Department of Neurobiology, Care Sciences and Society, Karolinska Institutet, Stockholm, Sweden

^6^Faculty of Medicine, University of Southampton, Southampton, United Kingdom

^7^Section of Allergy & Immunology, School of Medicine, University of Colorado Denver, Children’s Hospital Colorado, Anschutz Medical Campus, 13123 East 16th Avenue, Aurora, CO 80045, United States

^8^Department of Experimental Immunology and Department of Otorhinolaryngology, Amsterdam University Medical Centers, location AMC, Amsterdam, the Netherlands

^9^Division of Allergy and Immunology, Department of Dermatology, Allergy and Venerology, Charité Universitätsmedizin Berlin, Berlin, Germany

^10^Department of Pediatrics, OLVG Hospital, PO Box 95500, Amsterdam 1090HM, The Netherlands

^11^Rijnstate Allergy Centre, Rijnstate Hospital, PO Box 9555, Arnhem 6800TA, The Netherlands

^12^Usher Institute, University of Edinburgh, Edinburgh, United Kingdom

^13^Department of Mother and Child Health, The Referral Centre for Food Allergy Diagnosis and Treatment Veneto Region, University of Padua, Padua, Italy

^14^David Hide Asthma and Allergy Centre, St Mary’s Hospital, Isle of Wight, United Kingdom

^15^Wallenberg Centre for Molecular and Translational Medicine, University of Gothenburg, Sweden

**Correspondence:**

Bright Nwaru, PhD

Krefting Research Centre

Institute of Medicine

University of Gothenburg

Sweden

bright.nwaru@gu.se

**Supporting Information**

**1. Supplementary Figure S1.** Quality assessment of studies reporting on FAs outside the eight big FAs between September 2012 and June 2021

**2. Supplementary Figure S2.** Geographical distribution of frequently reported FAs outside the eight big FAs in Europe between September 2012 and June 2021

**3. Supplementary Table S1**. Summary of evidence on the frequency of allergy/sensitization to fruit, vegetables/legumes, and herbs/condiments/seeds/spices in Europe from studies published between September 2012 and June 2021

**4.Supplementary Table S2.** Summary of evidence on the frequency of food allergy/sensitization to cereals, meat, and other foods in Europe from studies published between September 2012 and June 2021

**5.** **Box S1.** Comparison between pooled estimates obtained for FA/FS outside the eight big foods and pooled estimates obtained for the eight big FA/FS between September 2012 and June 2021

**Supplementary Figures**

**Supplementary Figure S1.** Quality assessment of studies reporting on FAs outside the eight big FAs between September 2012 and June 2021

**Supplementary Figure S2.** Geographical distribution of frequently reported FAs outside the eight big FAs in Europe between September 2012 and June 2021

**Legend:** Vertical axis represents the number of studies reporting on each food. Horizontal axis displays all foods analysed.

**Notes:** Three studies were multi-centre studies, presenting data from more than one European country (i.e., European regions).

**Supplementary Tables**

**Supplementary Table S1.** Summary of evidence on the frequency of allergy/sensitization to fruit, vegetables/legumes, and herbs/condiments/seeds/spices in Europe from studies published between September 2012 and June 2021

| **Reference, country** | **Estimates of the frequency of fruit allergy/sensitization**  **Percentage (95% CI)** | **Estimates of the frequency vegetables/legumes allergy/sensitization**  **Percentage (95% CI)** | **Estimates of the frequency of herbs/condiments/seeds/spices allergy/sensitization**  **Percentage (95% CI)** | **Comments** |
| --- | --- | --- | --- | --- |
| Bröms et al. 2013, Sweden^7^ | Point prevalence of SR FA in children:  - stone fruit allergy:  - 1-2 years old: 0.8% (0.42-1.52)  - 3-4 years old: 1% (0.65-1.54)  - 5-6 years old: 1.35% (0.91-2.01) |  |  | The authors also investigated on cow’s milk, egg, soy, fish, peanuts, and nuts allergy/sensitization |
| Burney et al. 2014, Le et.al., 2015, Lyons et al. 2019, Switzerland, Spain, The Netherlands, Poland, Bulgaria, Greece, Lithuania, Iceland ^8-10^ | Data available from Burney et al. 2014:  Point prevalence of sIgE sensitization in adults (20-54 years old):  • in Europe:  - apple: 6.5%  - banana: 3.79%  - kiwi: 5.2%  - melon: 3.10%  - peach: 7.9%  - tomato: 4.9%  DBPCFC-verified FA:  • in Europe:  - apple 0.05% (0.02-0.09)  - peach 0.03% (0.01-0.07)  Data available from Le et al. 2015:  Point prevalence of SR FA:  • in The Netherlands:  - apple 2.35% (1.92-2.88)  - banana 0.35% (0.21-0.59)  - carrot 0.45% (0.28-0.72)  - kiwi 1.2% (0.9-1.6),  - melon 0.3% (0.17-0.52)  - peach 0.25% (0.13-0.46)  - tomato 0.7% (0.48-1.01)  DBPCFC-verified FA:  • in The Netherlands:  - apple 0.13% (0.06-0.3)  - peach 0.03% (0-0.15)  Data available form Lyons et al. 2019:  Point prevalence symptoms + sIgE positivity:  • in Switzerland  - apple 1.90% (0.99-3.15)  - banana 0.05% (0.02-0.42)  - kiwi 1.34% (0.60-2.42)  - melon 0.46% (0.09-1.17)  - peach: 2.02% (1.08-3.30)  - tomato 0.10% (0.00-0.55);  • in Spain  - apple 0.57% (0.08-1.65)  - banana 0.04% (0.00-0.65)  - kiwi 0.64% (0.11-1.77)  - melon 0.95% (0.25-2.24)  - peach 1.61% (0.63-3.18)  - tomato 0.15% (0.01-0.90)  • in Iceland  - apple 0.05% (0.02-0.48)  - banana 0.49% (0.08-1.29)  - kiwi 0.31% (0.02-0.99)  - melon 0.10% (0.00-0.60)  - peach 0.00% (0.00-0.28)  - tomato 0.15% (0.01-0.71)  • in Poland  - apple 0.75% (0.17-1.83)  - banana 0.00% (0.00-0.36)  - kiwi 0.30% (0.01-1.09)  - lentil 0.00% (0.00-0.36)  - melon 0.00% (0.00-0.36)  - peach 0.45 % (0.05-1.35)  - tomato 0.30% (0.01-1.10)  • in The Netherlands  - apple 0.91% (0.34-1.77)  - banana 0.05% (0.01-0.39),  - kiwi 0.57% (0.15-1.29)  - melon 0.05% (0.01-0.39)  - peach 0.60 % (0.17-1.33)  - tomato 0.08% (0.01-0.46)  • in Greece  - apple 0.00% (0.00-0.68)  - banana 0.00% (0.00-0.68)  - kiwi 0.00% (0.00-0.68)  - melon 0.00% (0.00-0.68)  - peach 0.10% (0.00-1.14)  - tomato 0.00% (0.00-0.68) | Data available from Burney et al. 2014:  Point prevalence of sIgE sensitization in adults (20-54 years old):  • in Europe:  - celery: 6.2%  - carrot: 6.0%  - lentils: 2.88%  Data available from Le et al. 2015:  Point prevalence SR FA in The Netherlands:  - carrot 0.45% (0.28-0.72)  DBPCFC-verified FA:  • in The Netherlands:  - celery 0.05% (0.01-0.19)  Data available form Lyons et al. 2019:  Point prevalence symptoms + sIgE positivity:  • in Switzerland  - carrot 1.01% (0.39-1.97)  - celery 0.24% (0.01-0.81)  - lentil 0.16% (0.07-0.93)  • in Spain  - carrot 0.81% (0.18-2.03)  - celery 0.00% (0.00-0.44)  - lentil 0.00% (0.00-0.44)  • in Iceland  - carrot 0.38% (0.05-1.12)  - celery 0.33% (0.03-1.03)  - corn 0.05% (0.02-0.48)  - lentil 0.00% (0.00-0.28)  - mustard seed 0.00% (0.00-0.28)  • in Poland  - celery 0.07% (0.02-0.63)  - carrot 0.15% (0.00-0.80)  - lentil 0.00% (0.00-0.36)  • in The Netherlands  - carrot 0.23% (0.01-0.76)  - celery 0.03 (0.03-0.32)  - lentil 0.03% (0.03-0.32)  • in Greece  - carrot 0.00% (0.00-0.68)  - celery 0.00% (0.00-0.68)  - lentil 0.00% (0.00-0.68) | Data available from Burney et al. 2014:  Point prevalence of sIgE sensitization in adults (20-54 years old):  • in Europe:  - buckwheat 2.90%  - mustard seed: 1.60%  - poppy seed: 3.03%  - sesame seed: 4.5%  - sunflower seed: 3.17%  Data available form Lyons et al. 2019:  Point prevalence symptoms + sIgE positivity:  • in Switzerland  - mustard seed 0.00% (0.00-0.25)  - sesame seed 0.03% (0.00-0.37)  - sunflower seed 0.05% (0.02-0.42)  - poppy seed 0.00% (0.00-0.25)  • in Spain  - mustard seed 0.00% (0.00-0.44)  - sesame seed 0.00% (0.00-0.44)  - sunflower seed 0.00% (0.00-0.44)  - poppy seed 0.00% (0.00-0.44)  • in Iceland  - mustard seed 0.00% (0.00-0.28)  - sunflower seed 0.00% (0.00-0.28)  - sesame seed 0.00% (0.00-0.28)  - poppy seed0.00% (0.00-0.28)  • in Poland  - mustard seed 0.00% (0.00-0.52)  - sunflower seed 0.00% (0.00-0.36)  - sesame seed 0.00% (0.00-0.36  - poppy seed 0.00% (0.00-0.52)  • in The Netherlands  - mustard - seed 0.00% (0.00-0.21)  - sesame seed 0.00% (0.00-0.21)  - sunflower seed 0.00% (0.00-0.21)  - poppy seed 0.00% (0.00-0.21)  • in Greece  - mustard seed 0.00% (0.00-0.68)  - sesame seed 0.00% (0.00-0.68)  - sunflower seed 0.07% (0.17-1.05)  - poppy seed 0.00% (0.00-0.68) | The authors also reported on cow’s milk, hen’s egg, wheat, soy, peanut, tree nut, fish, and shellfish, corn allergy/sensitization.  sIgE point prevalence was estimated as the prevalence of those with a specific IgE response to a particular food among ‘cases’ and ‘controls’ weighted back to the general population according to the sampling fraction by which these had been selected for further study. Since the sampling fraction was not mentioned by the authors, it was not possible to define precise confidence intervals for meta-analysis. Therefore, data for sIgE positivity have not been included in meta-analysis.  For Lithuania and Greece data on sIgE sensitization were not available.  In Lyons et al. 2019, the data on population prevalence estimation were obtained by the authors using a weighting procedure fully explained in the paper online repository.  DBPCFC was employed to assess FA to hazelnut, peanut, apple, peach, celery, shrimps. No challenges were performed for the other foods investigated |
| Clausen et al. 2017, Sweden^11^ | Point prevalence SR physician- diagnosed FA in children:  - stone fruits: 1.62% (1.26-2.09) | Point prevalence SR physician- diagnosed FA:  - peas: 0.3% (0.17-0.54) |  | The authors also investigated on cow’s milk, egg, soy, fish, peanuts, tree nuts, and fish allergy/sensitization  Data from Clausen et al 2017 were extracted from a thesis. The data here reported were extrapolated by reporting the sum of possible FA and probable FA in table 2 of the manuscript. |
| De Jong et al. 2019, The Netherlands^12^ | Point prevalence of SPT sensitization in children:  - peach: 3.8% (3.26-4.43) |  |  | The authors also investigated on peanuts and tree nuts allergy/sensitization |
| Dereci et al. 2015, Haktanir et al. 2017, Turkey ^13-14^ | Lifetime prevalence SR FA in children:  - blueberry: 0.00051% (0.00016-0.00086)  - kiwi allergy: 0.46% (0.36-0.57)  Point prevalence of SPT/PTP sensitization in children:  - blueberry: 0.02% (0.01-0.06)  - kiwi allergy: 0.11% (0.07-0.17)  Point prevalence of OFC/DBPCFC-verified FA in children:  - blueberry: 0.0000634% (0-0.001877)  - kiwi allergy 0.1% (0.06-0.16) |  |  | The authors also investigated tree nuts allergy/sensitization. Data were published in a different paper. |
| Doğruel et al. 2016, Turkey^15^ | Cumulative incidence of SPT and/or sIgE sensitization:  - banana: 0.11% (0.02-0.61)  Cumulative incidence of SPT sensitization:  - banana: 0.11% (0.02-0.61)  Cumulative incidence s-IgE sensitization:  - banana: 0%  Cumulative incidence of OFC-verified FA  - banana: 0.07% (0.01-0.41) |  |  | The authors also investigated on cow’s milk, hen’s egg, soy, peanut, fish, chicken, beef allergy/sensitization. |
| Gaspar -Marques et al. 2014, Portugal^16^ | Point prevalence SR FA in children:  - kiwi: 0.58% (0.28-1.18)  - orange: 0.49% (0.23-1.07)  - peach: 0.25% (0.08-0.72)  - strawberry: 2.30 % (1.60-3.31) |  |  | The authors also investigated on cow’s milk, egg, wheat, soy, peanut, tree nuts, fish, shellfish, chocolate allergy/sensitization |
| Grabenhenrich et al. 2020,  Iceland, United Kingdom, The Netherlands, Germany, Poland, Lithuania, Spain and Greece^17^ | Lifetime prevalence SR FA:  • in Europe  - apple: 0.36% (0.24-0.55)  - citrus fruits, not specified: 0.41% (0.28-0.61)  - kiwi: 0.46% (0.32-0.67)  - strawberry: 0.44% (0.31-0.65)  - tomato: 0.54% (0.39-0.76)  • in Iceland:  - apple: 0.11% (0.02-0.6)  - citrus fruits, not specified: 0.21% (0.06-0.77)  - kiwi: 0.32% (0.11-0.93)  - strawberry: 0%  - tomato: 0.32% (0.11-0.93)  • in the United Kingdom  - apple: 0.22% (0.04-1.24)  - citrus fruits, not specified: 0.22% (0.04-1.24)  - kiwi: 0.44% (0.12-1.59)  - strawberry: 0.66% (0.22-1.92)  - tomato: 1.76% (0.9-3.44)  • in The Netherlands  - apple: 0.31% (0.08-1.11)  - citrus fruits, not specified: 0.15% (0.03-0.86)  - kiwi: 0.92% (0.42-1.99)  - strawberry: 0.61% (0.24-1.57)  - tomato: 1.23% (0.62-2.4)  • in Germany  - apple: 0.7% (0.34-1.44)  - citrus fruits, not specified: 0.5% (0.21-1.16)  - kiwi: 0.2% (0.05-0.73)  - tomato: 0.5% (0.21-1.16  - strawberry: 0.9% (0.47-1.7)  • in Poland  - apple: 0.98% (0.5-1.92)  - citrus fruits, not specified: 1.22% (0.66-2.23)  - kiwi: 0.85% (0.41-1.75)  - strawberry: 1.1% (0.58-2.08)  - tomato: 0.73% (0.34-1.59  • in Lithuania  - apple: 0.32% (0.11-0.93)  - citrus fruits, not specified: 0.63% (0.29-1.37)  - kiwi: 0.11% (0.02-0.59)  - strawberry: 0.21% (0.66-0.77)  - tomato: 0.32% (0.11-0.93)  • Spain  - apple: 0%  - citrus fruits, not specified: 0%  - kiwi: 1.02% (0.49-2.09)  - strawberry: 0%  - tomato: 0%  • in Greece  - kiwi: 0%  - apple: 0%  - citrus fruits, not specified: 0%  - tomato: 0%  - strawberry: 0%  Lifetime prevalence of self-reported physician-diagnosed FA:  • In Europe  - kiwi: 0.18% (0.1-0.32)  - apple: 0.15% (0.08-0.28)  - citrus fruits, not specified: 0.13% (0.07-0.26)  - strawberry: 0.12% (0.06-0.24)  - tomato: 0.13% (0.07-0.26)  • in Iceland  - apple: 0%  - citrus fruits, not specified: 0%  - kiwi: 0.21% (0.06-0.77)  - strawberry: 0%  - tomato: 0%  • in the United Kingdom  - apple: 0%  - citrus fruits, not specified: 0%  - kiwi: 0%  - strawberry: 0.22% (0.04-1.24)  - tomato: 0.22% (0.04-1.24)  • in The Netherlands  - apple: 0%  - citrus fruits, not specified: 0%  - kiwi: 0.15% (0.03-0.86)  - tomato: 0.15% (0.03-0.86)  - strawberry: 0%  • in Germany  - apple: 0%  - citrus fruits, not specified: 0%  - kiwi: 0.1% (0.02-0.56)  - strawberry: 0.2% (0.05-0.73)  - tomato: 0%  • in Poland  - apple: 0.73% (0.34-1.59)  - citrus fruits, not specified: 0.73% (0.34-1.59)  - kiwi: 0.37% (0.12-1.07)  - strawberry: 0.49% (0.19-1.25)  - tomato: 0.49% (0.19-1.25)  • in Lithuania  - apple: 0.32% (0.11-0.93)  - citrus fruits, not specified: 0.21% (0.66-0.77]);  - kiwi: 0%  - strawberry: 0%  - tomato: 0.21% (0.66-0.77)  • in Spain  - apple: 0%  - citrus fruits, not specified: 0%  - kiwi: 0.58% (0.23-1.49)  - strawberry: 0%  - tomato: 0%  • in Greece  - apple: 0%  - citrus fruits, not specified: 0%  - kiwi: 0%  - strawberry: 0%  - tomato: 0% |  |  | The authors investigated also on cow’s milk, egg, wheat, soy, peanut, tree nuts, fish, shellfish allergy/sensitization.  For other FA to specific allergens with SPT positivity data are presented in a bar graph (Figure 3). |
| Grimshaw et al. 2016, United Kingdom^18^ |  | Cumulative incidence of FA by positive DBPCFC or clinical history  - lentil: 0.1% (0–0.32)  - broccoli: 0.1% (0–0.32) |  | The authors investigated also on cow’s milk, egg, wheat, soy, peanut, fish allergy/sensitization |
| Haftenberger et al. 2013, Germany^19^ | Point prevalence of sIgE sensitization in adults:  - apple: 9.2% (8.2-10.2)  - cherries: 10.1% (9.2-11.1)  - kiwi: 7.5% (6.7-8.4)  - peach: 12.4% (11.4-13.6)  - strawberry: 5.5% (4.8-6.2)  - tomato: 6.1% (5.5-6.9) | Point prevalence of sIgE sensitization in adults:  - carrot: 8.7% (7.8-9.5)  - celery: 8.6% (7.8-9.4)  - lupins: 3.9% (3.4-4.5)  - potato: 5.05% (4.5-5.6) | Point prevalence of sIgE sensitization in adults:  - sesame: 6.5% (5.8-7.1) | The authors investigated also cow’s milk, wheat, soy, peanut, tree nuts, shellfish, chicken, rye, barley, rice allergy/sensitization |
| Ivakhnenko et al. 2013, Ukraine^21^ | Lifetime prevalence of SR FA in children:  - citrus 18.1 (15.84-20.61)  - fruit: 9.3% (7.65-11.26) | Lifetime prevalence of SR FA in children:  - vegetables: 4.7% (3.55-6.19) |  | The author reported about cow’s milk, hen’s egg, fish  and others FA |
| Järvenpää et al., 2014, Finland^22^ | Point prevalence of SR FA in children:  - fruits and vegetables: 3.2% (2.5–4.2) | Point prevalence of SR FA in children:  - legumes: 0.8% (0.5–1.4)  - fruits and vegetables: 3.2% (2.5–4.2) | Point prevalence of SR FA in children:  - spices: 0.5% (0.3–1.0) | The authors also investigated on cow’s milk, egg, wheat, soy, and fish allergy/sensitization |
| Jurado-Escobar et al. 2017, Spain^23^ | Point prevalence SPT sensitization:  - peach: 2.1% (1.47-2.99) |  |  | Vegetal allergy included both fruit and vegetables allergy |
| Kaya et al. 2013, Turkey^24^ | Lifetime prevalence SR FA:  - tomato 1.2% (1.01-1.43)  Point prevalence SPT sensitization:  - kiwi: 0.01% (0-0.06)  - banana: 0.01% (0-0.06)  Point prevalence sIgE sensitization:  - strawberry: 0.01% (0-0.06)  - kiwi: 0.01% (0-0.06)  - banana: 0.01% (0-0.06)  OFC-verified FA:  - tomato: 0.01% (0-0.06)  DBPCFC-verified FA:  - tomato 0.01% (0-0.06)  - kiwi 0.01% (0-0.06)  - banana: 0.01% (0-0.06) |  | Point prevalence of SPT sensitization:  - sesame: 0.01% (0-0.06)  Point prevalence of sIgE sensitization:  - black pepper: 0.01% (0-0.06)  OFC-verified FA:  - sesame: 0.01% (0-0.06) | The authors also investigated on cow’s milk, egg, tree nuts peanut allergy/sensitization |
| Lyons et al. 2020, The Switzerland, Spain, Iceland, Lithuania, Bulgaria, Poland, Netherlands, Greece^25^ | Point prevalence of sIgE sensitization:  • in Switzerland:  - apple: 11.95 (10.60-13.30)  - banana: 15.46 (13.96-16.96)  - kiwi: 9.49 (8.27-10.71)  - melon: 9.19 (7.99-10.39)  - peach: 13.21 (11.80-14.62)  - tomato: 13.27 (11.86-14.68)  • in Spain  - apple: 10.13 (8.50-11.76)  - banana: 15.01 (13.09-16.94)  - kiwi: 9.22 (7.66-10.78)  - melon: 6.70 (5.35-8.05)  - peach: 12.06 (10.30-13.81)  - tomato: 9.00 (7.46-10.54  • in Greece  - apple: 4.85 (3.85-5.86)  - banana: 9.48 (8.11-10.85)  - kiwi: 5.10 (4.08-6.13)  - melon: 3.40 (2.56-4.25)  - peach: 6.81 (5.63-7.98)  - tomato: 4.85 (3.85-5.86)  • in The Netherlands  - apple: 7.90 (6.77-9.04)  - banana: 8.93 (7.73-10.14)  - kiwi: 9.53 (8.29-10.77)  - melon: 4.26 (3.41-5.12)  - peach: 8.30 (7.14-9.47)  - tomato: 6.09 (5.08-7.10)  • in Lithuania  - apple: 4.93 (4.14-5.72)  - banana: 7.21 (6.27-8.16)  - kiwi: 4.54 (3.78-5.31)  - melon: 2.28 (1.73-2.82)  - peach: 5.68 (4.83-6.53)  - tomato: 3.79 (3.09-4.49)  • in Poland  - apple: 5.50 (4.50-6.50)  - banana: 6.05 (5.00-7.10)  - kiwi: 5.37 (4.38-6.36  - melon: 3.99 (3.13-4.86)  - peach: 6.46 (5.38-7.54)  - tomato: 5.09 (4.13-6.06)  • in Iceland  - apple: 2.05 (1.46-2.64)  - banana: 3.04 (2.33-3.75)  - kiwi: 1.74 (1.20-2.28)  - melon: 0.81 (0.44-1.18)  - peach: 2.49 (1.84-3.13)  - tomato: 2.55 (1.90-3.23)  Point prevalence symptoms + sIgE sensitization:  • in Switzerland:  - apple: 0.54 (0.02-1.80)  - banana: 0.14 (0.04-0.98)  - kiwi: 0.27 (0.00-1.29) Greece:  - melon: 0.00 (0.00-0.49)  - peach: 0.14 (0.04-0.98)  - tomato: 0.27 (0.00-1.29)  • in Spain  - apple: 0.18 (0.02-1.15)  - banana: 0.18 (0.02-1.15)  - kiwi: 1.06 (0.19-2.74)  - melon: 0.18 (0.02-1.15)  - peach: 1.06 (0.19-2.74)  - tomato: 0.35 (0.00-1.52)  • in Greece  - apple: 0.00 (0.00-0.88  - banana: 0.56 (0.00-2.51)  - kiwi: 0.00 (0.00-0.88)  - melon: 0.00 (0.00-0.88)  - peach: 0.28 (0.07-1.89)  - tomato: 0.28 (0.07-1.89)  • in The Netherlands  - apple: 0.84 (0.18-2.05)  - banana: 0.32 (0.01-1.18)  - kiwi: 0.63 (0.09-1.72)  - melon: 0.00 (0.00-0.37)  - peach: 0.53 (0.06-1.55)  - tomato: 0.11 (0.02-0.74)  • in Lithuania  - apple: 0.89 (0.01-3.17)  - banana: 0.00 (0.00-0.86)  - kiwi: 0.44 (0.02-2.28)  - melon: 0.00 (0.00-0.86)  - peach: 0.44 (0.02-2.28)  - tomato: 0.00 (0.00-0.86)  • in Poland  - apple: 1.09 (0.32-2.38)  - banana: 0.95 (0.25-2.18)  - kiwi: 0.31 (0.01-1.14)  - melon: 0.00 (0.00-0.37)  - peach: 0.48 (0.05-1.43)  - tomato: 0.63 (0.10-1.68)  • in Iceland  - apple: 0.07 (0.03-0.63)  - banana: 0.07 (0.03-0.63)  - kiwi: 0.15 (0.01-0.80)-  - melon: 0.00 (0.00-0.35)  - peach: 0.00 (0.00-0.35)  - tomato: 0.07 (0.03-0.63) | Point prevalence of sIgE sensitization:  • in Switzerland:  - carrot: 12.46 (11.09-13.83)  - celery: 13.09 (11.69-14.49)  - lentils: 7.99 (6.86-9.11)  • in Spain  - carrot: 8.36 (6.87-9.85)  - celery: 8.09 (6.62-9.56)  - lentils: 7.45 (6.04-8.87)  • in Greece  - carrot: 4.85 (3.85-5.86)  - celery: 3.88 (2.98-4.79)  - lentils: 3.63 (2.76-4.51)  • in The Netherlands  - carrot: 7.20 (7.11-8.29)  - celery: 6.89 (5.83-7.96)  - lentils: 5.38 (4.43-6.33)  )  • in Lithuania  - carrot: 4.54 (3.78-5.31)  - celery: 4.93 (4.13-5.71)  - lentils: 2.65 (2.06-3.24)  • in Poland  - carrot: 5.09 (4.13-6.06)  - celery: 5.78 (4.76-6.81)  - lentils: 3.99 (3.13-4.85)  • in Iceland  - apple: 2.05 (1.46-2.64)  - banana: 3.04 (2.33-3.75)  - buckwheat: 1.37 (0.89-1.85)  - carrot: 2.11 (1.52-2.71)  - lentils: 2.11 (1.52-2.71)  Point prevalence symptoms + sIgE sensitization:  • in Switzerland:  - carrot:0.81 (0.10-2.27)  - celery: 0.14 (0.04-0.98)  - lentils: 0.00 (0.00-0.49)  • in Spain  - carrot: 0.00 (0.00-0.56)  - celery: 0.00 (0.00-0.56)  - lentils: 0.53 (0.02-1.85)  • in Greece  - carrot: 0.00 (0.00-0.88)  - celery: 0.00 (0.00-0.56)  - lentils: 0.56 (0.00-2.51)  • in The Netherlands  - carrot: 0.11 (0.02-0.74)  - celery: 0.00 (0.00-0.37)  - lentils: 0.00 (0.00-0.37)  • in Lithuania  - carrot: 0.89 (0.01-3.17)  - celery: 0.00 (0.00-0.86)  - lentils: 0.00 (0.00-0.86)  • in Poland  - carrot: 0.15 (0.01-0.83)  - celery: 1.24 (0.40-2.60)  - lentils: 0.00 (0.00-0.37)  • in Iceland  - carrot: 0.00 (0.00-0.35)  - carrot: 0.00 (0.00-0.35)  - celery: 0.00 (0.00-0.35)  - lentils: 0.00 (0.00-0.35) | Point prevalence of sIgE sensitization:  • in Switzerland:  - buckwheat: 8.89 (7.70-10.07)  - mustard seed: 4.92 (4.02-5.82)  - poppy seed: 8.50 (7.34-9.66)  - sesame seed: 12.10 (10.74-13.45)  - sunflower seed: 8.89 (7.70-10.07)  • in Spain  - buckwheat: 7.55 (6.13-8.98)  - mustard seed 3.33 (2.36-4.29)  - poppy seed: 6.32 (5.01-7.64)  - sesame seed: 11.90 (10.15-13.64)  - sunflower seed: 6.16 (4.87-7.46)  • in Greece  - buckwheat: 5.58 (4.51-6.66)  - mustard seed: 2.18 (1.50-2.86)  - poppy seed: 3.15 (2.34-3.97)  - sesame seed: 5.82 (4.73-6.92)  - sunflower seed: 4.85 (3.85-5.86)  • in The Netherlands  - buckwheat: 4.77 (3.87-5.67)  - mustard seed: 1.72 (1.17-2.27)  - poppy seed: 4.88 (3.97-5.78)  - sesame seed: 6.50 (5.46-7.54)  - sunflower seed: 4.67 (3.78-5.56)  • in Lithuania  - buckwheat: 2.65 (2.06-3.24)  - mustard seed: 3.04 (2.41-3.67)  - poppy seed: 2.28 (1.73-2.82)  - sesame seed: 3.03 (2.40-3.66)  - sunflower seed: 3.41 (2.75-4.08)  • in Poland  - buckwheat: 3.99 (3.13-4.85)  - mustard seed:2.48 (1.80-3.16)  - poppy seed: 3.99 (3.13-4.85)  - sesame seed: 5.50 (4.50-6.51)  - sunflower seed: 4.54 (3.63-5.46)  • in Iceland  - buckwheat: 1.37 (0.89-1.85)  - mustard seed: 0.37 (0.12-0.63)  - poppy seed: 0.75 (0.39-1.10)  - sesame seed: 2.86 (2.17-3.55)  - sunflower seed: 1.37 (0.89-1.85)  Point prevalence symptoms + sIgE sensitization:  • in Switzerland:  - buckwheat: 0.14 (0.04-0.98)  - mustard seed: 0.00 (0.00-0.49)  - poppy seed: 0.00 (0.00-0.49)  - sesame seed: 0.00 (0.00-0.49)  - sunflower seed: 0.00 (0.00-0.49)  • in Spain  - buckwheat: 0.00 (0.00-0.56)  - mustard seed 0.00 (0.00-0.56)  - poppy seed: 0.00 (0.00-0.56)  - sesame seed: 0.00 (0.00-0.56)  - sunflower seed: 0.53 (0.02-1.85)  • in Greece  - buckwheat: 0.00 (0.00-0.88)  - mustard seed: 0.00 (0.00-0.88)  - poppy seed: 0.00 (0.00-0.88)  - sesame seed: 0.00 (0.00-0.88)  - sunflower seed: 0.00 (0.00-0.88)  • in The Netherlands  - buckwheat: 0.00 (0.00-0.37)  - mustard seed: 0.00 (0.00-0.37)  - poppy seed: 0.00 (0.00-0.37)  - sesame seed: 0.00 (0.00-0.37)  - sunflower seed: 0.00 (0.00-0.37):  • in Lithuania  - buckwheat: 0.00 (0.00-0.86)  - mustard seed: 0.00 (0.00-0.86)  - poppy seed: 0.00 (0.00-0.86)  - sesame seed: 0.00 (0.00-0.86)  - sunflower seed: 0.00 (0.00-0.86)  - tomato: 0.00 (0.00-0.86)  • in Poland  - buckwheat: 0.00 (0.00-0.37)  - mustard seed: 0.00 (0.00-0.37)  - poppy seed: 0.00 (0.00-0.37)  - sesame seed: 0.00 (0.00-0.37)  - sunflower seed: 0.00 (0.00-0.37)  -  • in Iceland  - buckwheat: 0.07 (0.03-0.63)  - mustard seed: 0.00 (0.00-0.35)  - poppy seed: 0.00 (0.00-0.35)  - sesame seed: 0.15 (0.01-0.80)  - sunflower seed: 0.00 (0.00-0.35) | The authors also investigated on cow’s milk, egg, wheat, soy, peanut, tree nuts, fish, shellfish  Data on population prevalence estimation were obtained by the authors using a weighting procedure fully explained in the paper online repository. Data on DBPCFC were not reported in meta-analysis as the authors claim that the number of subjects who agreed to be tested was too low to infer a valid population prevalence estimate on confirmed FA by DBPCFC |
| Mortz et al. 2013, Denmark^26^ |  |  | Point prevalence sIgE sensitization to sesame 1.29% (0.59-2.78)  Point prevalence SPT sensitization to sesame 3.91% (2.49-6.1) |  |
| Mustafayev et al., 2013, Turkey^27^ | Point prevalence of OFC-verified FA:  - peach: 0.02% (0-0.1)  - kiwi: 0.02% (0-0.1) | Point prevalence of OFC-verified FA:  - spinach: 0.02% (0-0.1) |  | The authors also investigated on cow’s milk, egg, wheat, peanut, tree nuts, fish, beef and cheese allergy/sensitization. |
| Patelis et al. 2014, Sweden and Iceland^28^ | Lifetime prevalence of SR FA  - fruit: 9.32% (8.2-10.57) | Lifetime prevalence of SR FA  - vegetables: 4.2% (3.46-5.1) | Lifetime prevalence of SR FA  - chocolate: 1% (0.67-1.49)  - herbs, chilli, garlic: 0.87% (0.56-1.34) | The authors also investigated on cow’s milk, egg, wheat, peanut, tree nuts, fish, meat allergy/sensitization  peanut, hazelnut, fish, beef. |
| Raciborski et al 2012, Poland^29^ | Point prevalence of SR FA  - Fruit: 8.01% (6.84-9.35) |  |  | The authors also investigated on chocolate allergy/sensitization |
| Rentzos 2019 et al. Sweden^30^ | Point prevalence of SR FA:  - apple 8.4% (6.7–10.1)  - apricot 1.7% (0.9–2.5)  - avocado 0.85% (0.2–1.3)  - banana1.35 (0.6–2.0)  - carrot 3.15 (2.0–4.1)  - cherry 3.1% (2.0–4.1)  - kiwi 7.35 (5.7–8.8)  - lingonberry 0.1% (−0.1–0.3)  - melon 0.4% (0.0–0.8)  - nectarine 2.4% (1.5–3.3)  - orange 2.95% (1.9–3.9)  - parsley 0.3% (0.0–0.6)  - peas 0.7% (0.2–1.2)  - peach 3.25% (2.1–4.3)  - pear 4.0 (2.8–5.2)  - plum 3.0 (2.0–4.0)  - strawberry 2.7% (1.7-3.7)  - tomato 2.1% (1.2-3.0) | Point prevalence of SR FA:  - bean1.8% (1.0–2.6)  - celery 0.3 % (0.0–0.6)  - coriander 0.1% (−0.1–0.3)  - potato 1.6% (0.9–2.4)  - sweet pepper 2.1% (1.2-3.0) | Point prevalence of SR FA:  - anise/caraway 0.2% (0.1 - 0.5)  - cayenne/red pepper 1.6% (0.9–2.4)  - chili/tabasco 2.2% (1.3–3.1)  - poppy seed 0.1 (−0.1–0.3)  - sesame 0.1% (-0.1-03)  - sunflower 0.1% (-0.1-0.3) | The authors also investigated on cow’s milk, egg, wheat, soy, peanut, tree nuts, fish, shellfish, beef camomile, cheese, chicken, chocolate, dried fruit, non wheat flour, fried/fat food, pork, red meat, salami, allergy/sensitization. |
| Skypala et al. 2013, United Kingdom^31^ | Life-time prevalence of SR FA:  - non-citrus fruit: 4.7% (4.05-5.44)  - citrus fruit: 1.3% (0.98-1.73)  - tomato: 0.9% (0.64-1.27) | Life-time prevalence of SR FA:  - vegetables: 3.3% (2.76-3.94)  - beans and lentils: 0.5% (0.32-0.79) | Life-time prevalence of SR FA:  - curry and spices: 1.3 (0.98-1.73)  - seeds: 0.7% (0.48-1.03) | The authors also investigated on cow’s milk, egg, wheat, soy, peanut, fish, food additives, shellfish, allergy/sensitization. |
| Stefanaki et al. 2018 Greece^32^ | Life-time prevalence of SR FA:  -tomato 1.31% (0.75-2.27) |  |  | The authors also investigated on cow’s milk, egg, peanut, tree nuts, fish allergy/sensitization.  Data were extracted from a conference abstract |
| Strinnholm et al. 2014, Sweden^33^ | Point prevalence self-report FHS:  - fruits and nuts 14.58% (13.28-16.0)  - kiwi 8.05% (7.06-9.16)  - orange 4.53% (3.79-5.40)  - apple 3.87% (3.19-4.68)  - raw carrots 1.39% (1.01-1.92)  - banana 0.7% (0.44-1.10) | Point prevalence self-report FHS:  - raw carrots 1.39% (1.01-1.92) |  | The authors also investigated on cow’s milk, egg, wheat, soy, peanut, tree nuts, fish allergy/sensitization. |
| Venkataraman et al. 2017, United Kingdom^35^ | Point prevalence of SR FA (according to author’s restrictive criteria to define FA):  At 1-year:  - fruits 0.79% (0.43-1.45)  - tomato 0.16% (0.04-0.57)  At 2-year:  -fruits: 0.95% (0.53-1.69)  - tomato: 0.16% (0.04-0.57)  At 4-year:  - fruit: 0.74% (0.39-1.4)  - tomato 0.33% (0.13-0.84)  At 10-year-old:  - fruit: 0.29% (0.11-0.75)  - tomato: 0.29% (0.11-0.75)  At 18-year:  - fruit: 0.85% (0.48-1.52)  - kiwi: 0.23% (0.08-0.68)  - tomato: 0.39% (0.17-0.9) |  |  | The authors also investigated on cow’s milk, egg, wheat, soy, peanut, tree nuts, fish, shellfish allergy/sensitization.  Estimates given report FA according to restrictive criteria defined by authors Therefore, these estimates were not reported in meta-analysis. |
| Westerlaken-van Ginkel et al. 2020, The Netherlands^36^ | Point prevalence SR FA:  - apple: 2.50% (2.39-2.61),  - kiwi: 0.98% (0.92-1.05)  - strawberry: 0.30% (0.26-0.34)  - cherry: 0.22% (0.19-0.25)  - pear: 0.12% (0.18-0.24)  - peach: 0.20% (0.17-0.24)  - banana: 0.14% (0.12.017) |  | Point prevalence SR FA:  - sesame: 0.15% (0.13-0.18) | The authors also investigated on cow’s milk, egg, wheat, soy, peanut, tree nuts, fish, shellfish allergy/sensitization. |

**Legend**: CI= confidence interval; DBPCFC= double blind placebo-controlled food challenge; FA= food allergy; FC= food challenge; OFC= oral food challenge; sIgE= specific immunoglobulin E; SPT= skin prick test; SR= self-reported

**Additional notes:** Data were extracted from conference abstracts or poster in the following studies: Dereci et al. 2015; Jurado-Escobar et al. 2017; Stefanaki et al. 2018; Raciborski et al 2012; Mortz, et al. 2013. For Clausen et al. 2017, data were extracted from a university thesis.

**Supplementary Table S2.** Summary of evidence on the frequency of food allergy/sensitization to cereals, meat, and other foods in Europe from studies published between September 2012 and June 2021

| **Reference, country** | **Estimates of the frequency of cereal allergy/sensitization**  **Percentage (95% CI)** | **Estimates of the frequency of meat allergy/sensitization**  **Percentage (95% CI)** | **Estimates of the frequency of other food allergy/sensitization**  **Percentage (95% CI)** | **Comments** |
| --- | --- | --- | --- | --- |
| Burney et al. 2014, Le et.al., 2015, Lyons et al. 2019, Switzerland, Spain, The Netherlands, Poland, Bulgaria, Greece, Lithuania, Iceland^8-10^ | Data available from Burney et al. 2014:  Point prevalence of sIgE sensitization to corn in adults (20-54 years old):  • in Europe:3.38%  Data available form Lyons et al. 2019:  Point prevalence symptoms + sIgE positivity to corn:  • In Switzerland: 0.05% (0.02-0.42)  • in Spain: 0.00% (0.00-0.44)  • in Iceland: 0.05% (0.02-0.48)  • in Poland: 0.00% (0.00-0.36)  • in The Netherlands: 0.00% (0.00-0.21)  • in Greece: 0.00% (0.00-0.68) |  |  | The authors also reported on cow’s milk, hen’s egg, wheat, soy, peanut, tree nut, fish, and shellfish allergy/sensitization.  sIgE point prevalence was estimated as the prevalence of those with a specific IgE response to a particular food among ‘cases’ and ‘controls’ weighted back to the general population according to the sampling fraction by which these had been selected for further study. Since the sampling fraction was not mentioned by the authors, it was not possible to define precise confidence intervals for meta-analysis. Therefore, data for sIgE positivity have not been included in meta-analysis.  For Lithuania and Greece data on sIgE sensitization were not available.  In Lyons et al. 2019, the data on population prevalence estimation were obtained by the authors using a weighting procedure fully explained in the paper online repository.  DBPCFC was employed to assess FA to hazelnut, peanut, apple, peach, celery, shrimps. No challenges were performed for the other foods investigated |
| Doğruel et al. 2016, Turkey^15^ |  | Cumulative incidence of SPT and/or s-IgE sensitization:  - chicken: 0.22% (0.06-0.79])  - beef: 0.22% (0.06-0.79)  Cumulative incidence SPT sensitization  - chicken: 0.22% (0.06-0.79)  - beef: 0.11% (0.02-0.61)  Cumulative incidence of sIgE sensitization to:  - chicken meat: 0.11% (0.02-0.61)  - beef: 0.22% (0.06-0.79)  Cumulative incidence of OFC-verified FA  - chicken: 0.07% (0.01-0.41) |  | The authors also investigated on cow’s milk, hen’s egg, soy, peanut, fish, banana allergy/sensitization. |
| Gaspar -Marques et al. 2014, Portugal^16^ |  |  | Point prevalence SR FA:  - chocolate: 1.40% (0.86-2.23) | The authors also investigated on cow’s milk, egg, wheat, soy, peanut, tree nuts, fish, shellfish, kiwi, orange, peach, strawberry allergy/sensitization |
| Haftenberger et al. 2013, Germany^19^ | Point prevalence of sIgE sensitization in adults:  - rye flour: 6.0% (5.4-6.7)  - barley flour: 6.3 (5.6-7.0)  - rice: 4.0% (3.5-4.6) | Point prevalence of sIgE sensitization in adults:  - chicken protein: 1.6% (1.3-2.0) |  | The authors investigated also cow’s milk, wheat, soy, peanut, tree nuts, shellfish, apple, cherry, kiwi, peach, carrot, celery, lupins, potato, sesame allergy/sensitization |
| Hicke-Roberts et al., 2020, Sweden^20^ | Cumulative incidence of SR FA (or intolerance) by 8 years: 11.7% (9.9-13.8)  - cereals: 0.7% (0.34-1.45) |  |  | The outcome investigated was SR FA or intolerance. The author also investigated on cow’s milk, egg, wheat, , peanut, tree nuts, and fish allergy/sensitization |
| Ivakhnenko et al. 2013, Ukraine^21^ |  |  | Lifetime prevalence of SR FA in children:  - chocolate: 12.3% (10.41-14.48) | The author also investigated on cow’s milk, hen’s egg, fish, citrus, fruit, vegetables allergy/sensitization |
| Kaya et al. 2013, Turkey^24^ |  |  | Lifetime prevalence SR FA:  - chocolate 3.5% (3.16-3.88)  Point prevalence of SPT sensitization:  - chocolate: 0.01% (0-0.06)  - honey: 0.01% (0-0.06)  Point prevalence of sIgE sensitization:  - honey: 0.01% (0-0.06)  DBPCF- verifies FA:  - chocolate: 0.01% (0-0.06)  - honey: 0.01% (0-0.06) | The authors also investigated on cow’s milk, egg, tree nuts peanut, kiwi, banana, strawberry, tomato, sesame, back pepper allergy/sensitization |
| Lyons et al. 2020, The Switzerland, Spain, Iceland, Lithuania, Bulgaria, Poland, Netherlands, Greece^25^ |  |  | Point prevalence of sIgE sensitization to corn:  • in Switzerland: 8.35 (7.20-9.50)  • in Spain: 9.43 (7.85-11.01)  • in Greece: 5.82 (4.73-6.92)  • in The Netherlands: 6.09 (5.08-7.10)  • in Lithuania: 3.04 (2.41-3.67)  • in Poland: 3.86 (3.01-4.70)  • in Iceland: 1.80 (1.25-2.35)  - mustard seed: 0.37 (0.12-0.63)  Point prevalence symptoms + sIgE sensitization:  • in Switzerland: 0.00 (0.00-0.49)  • in Spain: 0.18 (0.02-1.15)  • in Greece: 0.00 (0.00-0.88)  • in The Netherlands: 0.00 (0.00-0.86)  • in Lithuania: 0.00 (0.00-0.37)  • in Poland: 0.00 (0.00-0.37)  • in Iceland: 0.00 (0.00-0.35) | The authors also investigated on cow’s milk, egg, wheat, soy, peanut, tree nuts, fish, shellfish, fruit, vegetables, seed allergy/sensitization  Data on population prevalence estimation were obtained by the authors using a weighting procedure fully explained in the paper online repository.  Data on DBPCFC were not reported in meta-analysis as the authors claim that the number of subjects who agreed to be tested was too low to infer a valid population prevalence estimate on confirmed FA by DBPCFC |
| Mustafayev et al., 2013, Turkey^27^ |  | Point prevalence of OFC-verified FA:  - beef: 0.04% (0.01-0.13) | Point prevalence of OFC-verified FA:  - cheese: 0.02% (0-0.1) | The authors also investigated on cow’s milk, egg, wheat, peanut, tree nuts, fish, spinach, kiwi, peach allergy/sensitization. |
| Patelis et al. 2014, Sweden and Iceland^28^ |  | Lifetime prevalence of SR FA  - meat: 0.82% (0.53-1.28) |  | The authors also investigated on cow’s milk, egg, wheat, peanut, tree nuts, fish, fruit, vegetables, herbs/chilli/garlic, chocolate allergy/sensitization  peanut, hazelnut, fish, beef. |
| Raciborski et al 2012, Poland^29^ |  |  | Point prevalence SR FA  - Chocolate: 10.11% (8.8-11.59) | The authors also investigated on fruit allergy/sensitization |
| Rentzos 2019 et al. Sweden^30^ |  | Point prevalence of SR FA:  - beef 0.4 (0.0–0.8)  - chicken 0.1% (−0.1–0.3)  - pork 0.6% (0.1–1.0)  - red meat 0.9% (0.3–1.4)  - salami 0.5% (0.1–0.9) | Point prevalence of SR FA:  - camomile 0.6 (0.1–1.0)  - cheese: 1.8% (1.0–2.6)  - cheese (lactose intolerance symptom excluded): 0.8% (0.0–0.0)  - chocolate1.6% (0.9–2.4)  - dried fruit 0.3% (0.0–0.6)  - flour (non wheat): 0.7% (0.2–1.2)  - fried/fat food 3.7% (2.5–4.8) | The authors also investigated on cow’s milk, egg, wheat, soy, peanut, tree nuts, fish, shellfish, fruit, vegetables, seeds/herbs/condiments allergy/sensitization. |
| Skypala et al. 2013, United Kingdom^31^ |  |  | Life-time prevalence of SR FA:  - food additives: 1.61% (1.25-2.07 | The authors also investigated on cow’s milk, egg, wheat, soy, peanut, fish, citrus, non-citrus fruit, tomato, vegetables, beans and lentils, curry and spices, seeds, shellfish allergy/sensitization. |
| Topcu et al. 2019, Turkey^34^ |  | Point prevalence of SR FA beef allergy:  2.6% (2.19-3.08)  Point prevalence of DBPCFC- verified beef allergy:  0.30% (0.18-0.5) |  | The authors investigated also sIgE and SPT sensitization to beef but the data were not shared. |

**Legend**: CI= confidence interval; DBPCFC= double blind placebo-controlled food challenge; FA= food allergy; OFC= oral/open food challenge; sIgE= specific immunoglobulin E; SPT= skin prick test; SR= self-reported

**Additional notes:** Data were extracted from conference abstracts or poster in the following studies: Dereci et al. 2015; Raciborski et al 2012; Topcu et al. 2019. For Clausen et al. 2017, data were extracted from a university thesis. 2014, and Le et al. 2015.

**Box S1.** Comparison between pooled estimates obtained for FA/FS outside the eight big foods and pooled estimates obtained for the eight big FA/FS

**Legend.** SR= self-reported; FA= Food allergy; sIgE= specific-Immunoglobulin E.

**Additional notes:** the estimates on FA to the eight big foods are presented in *Italic*

Some of the studies were multi-centre studies, reporting multiple estimates/records for the same allergenic foods/outcome (i.e., one estimate/record from each country)

**References**

1. Nwaru BI, Hickstein L, Panesar SS, et al. The epidemiology of food allergy in Europe: a systematic review and meta-analysis. *Allergy.* 2014;69(1):62-75.

2. Nwaru BI, Hickstein L, Panesar SS, Roberts G, Muraro A, Sheikh A. Prevalence of common food allergies in Europe: a systematic review and meta-analysis. *Allergy.* 2014;69(8):992-1007.

3. Spolidoro GCI, Amera YT, Ali MM, et al. Frequency of food allergy in Europe: an updated systematic review and meta-analysis. *Allergy.* 2022;00(1398-9995 (Electronic)):1-18.

4. Spolidoro GCI, Ali MM, Amera YT, et al. Prevalence estimates of eight big food allergies in Europe: updated systematic review and meta-analysis [Manuscript submitted for publication]. In:2023.

5. Spolidoro GA-O, Lisik D, Nyassi S, et al. Prevalence of tree nut allergy in Europe: A systematic review and meta-analysis. LID - 10.1111/all.15905 [doi]. (1398-9995 (Electronic)).

6. Newcombe R. Two-sided confidence intervals for the single proportion: comparison of seven methods. . *Stat Med* 1998;17:857–872.

7. Bröms K, Norbäck D, Eriksson M, Sundelin C, Svärdsudd K. Prevalence and co-occurrence of parentally reported possible asthma and allergic manifestations in pre-school children. *BMC Public Health.* 2013;13:764.

8. Burney PG, Potts J, Kummeling I, et al. The prevalence and distribution of food sensitization in European adults. *Allergy.* 2014;69(3):365-371.

9. Le TM, van Hoffen E, Kummeling I, et al. Food allergy in the Netherlands: differences in clinical severity, causative foods, sensitization and DBPCFC between community and outpatients. *Clin Transl Allergy.* 2015;5:8.

10. Lyons SA, Burney PGJ, Ballmer-Weber BK, et al. Food Allergy in Adults: Substantial Variation in Prevalence and Causative Foods Across Europe. *J Allergy Clin Immunol Pract.* 2019;7(6):1920-1928 e1911.

11. Clausen I, Goksör E, Alm B, Wennergren G. *Food allergy at 12 years of age in Western Sweden – Risk factors and protective factors*, University of Iceland; 2017.

12. de Jong NW, Elbert NJ, Mensink-Bout SM, et al. Parental and child factors associated with inhalant and food allergy in a population-based prospective cohort study: the Generation R Study. *Eur J Pediatr.* 2019;178(10):1507-1517.

13. Dereci S, Orhan F, Koca T, Akcam M. Prevalence of blueberry allergy in a Turkish population. *Annals of Allergy, Asthma & Immunology.* 2015;114(0):259-260.

14. Haktanir Abul M, Dereci S, Hacisalihoglu S, Orhan F. Is kiwifruit allergy a matter in kiwifruit-cultivating regions? A population-based study. *Pediatr Allergy Immunol.* 2017;28(1):38-43.

15. Dogruel D, Bingol G, Altintas DU, Yilmaz M, Guneser Kendirli S. Clinical Features of Food Allergy during the 1st Year of Life: The ADAPAR Birth Cohort Study. *Int Arch Allergy Immunol.* 2016;169(3):171-180.

16. Gaspar-Marques J, Carreiro-Martins P, Papoila AL, et al. Food allergy and anaphylaxis in infants and preschool-age children. *Clin Pediatr (Phila).* 2014;53(7):652-657.

17. Grabenhenrich L, Trendelenburg V, Bellach J, et al. Frequency of food allergy in school-aged children in eight European countries-The EuroPrevall-iFAAM birth cohort. *Allergy.* 2020;75(9):2294-2308.

18. Grimshaw KE, Bryant T, Oliver EM, et al. Incidence and risk factors for food hypersensitivity in UK infants: results from a birth cohort study. *Clin Transl Allergy.* 2015;6:1.

19. Haftenberger M, Laussmann D, Ellert U, et al. [Prevalence of sensitisation to aeraoallergens and food allergens: results of the German Health Interview and Examination Survey for Adults (DEGS1)]. *Bundesgesundheitsblatt Gesundheitsforschung Gesundheitsschutz.* 2013;56(5-6):687-697.

20. Hicke-Roberts A, Wennergren G, Hesselmar B. Late introduction of solids into infants' diets may increase the risk of food allergy development. *BMC Pediatr.* 2020;20(1):273.

21. Ivakhnenko O, Nyankovskyy S. Nutritional status of babies and influence of unmodified cow's milk on allergic reactions according to the epidemiological study from Ukraine. *Pediatria Polska.* 2013;88(2):138-143.

22. Jarvenpaa J, Paassilta M, Salmivesi S, Sannisto T, Niitty S, Korppi M. Stability of parent-reported food allergy in six and 7-year-old children: the first 5 years of the Finnish allergy programme. *Acta Paediatr.* 2014;103(12):1297-1300.

23. Jurado-Escobar R, P erez-S anchez N, Victorio L, et al. Sensitisation and allergy patterns to inhalant and food allergens in a population from the Mediterranean area. *Allergy.* 2017;72:383-757.

24. Kaya A, Erkocoglu M, Civelek E, Cakir B, Kocabas CN. Prevalence of confirmed IgE-mediated food allergy among adolescents in Turkey. *Pediatr Allergy Immunol.* 2013;24(5):456-462.

25. Lyons SA, Knulst AC, Le T-M, et al. Prevalence of Food Sensitization and Food Allergy in Children Across Europe. *Journal of Allergy and Clinical Immunology: In Practice.* 2020;8(8):2736.

26. Mortz CG, Andersen KE, Bindslev-Jensen C. Allergy to sesame-prevalence in an unselected population and relation to pollen sensitisation. *Allergy: European Journal of Allergy and Clinical Immunology.* 2013;68(0):146

27. Mustafayev R, Civelek E, Orhan F, Yuksel H, Boz AB, Sekerel BE. Similar prevalence, different spectrum: IgE-mediated food allergy among Turkish adolescents. *Allergol Immunopathol (Madr).* 2013;41(6):387-396.

28. Patelis A, Gunnbjornsdottir M, Borres MP, et al. Natural history of perceived food hypersensitivity and IgE sensitisation to food allergens in a cohort of adults. *PLoS One.* 2014;9(1):e85333.

29. Raciborski F, Samel-Kowalik P, Tomaszewska A, et al. Food allergy in children aged 6-8 years in Poland. *Allergy: European Journal of Allergy and Clinical Immunology* 2012;67(0):610-611

30. Rentzos G, Johanson L, Goksor E, Telemo E, Lundback B, Ekerljung L. Prevalence of food hypersensitivity in relation to IgE sensitisation to common food allergens among the general adult population in West Sweden. *Clin Transl Allergy.* 2019;9:22.

31. Skypala IJ, Bull S, Deegan K, et al. The prevalence of PFS and prevalence and characteristics of reported food allergy; a survey of UK adults aged 18-75 incorporating a validated PFS diagnostic questionnaire. *Clin Exp Allergy.* 2013;43(8):928-940.

32. Stefanaki E, Margetaki A, Roumeliotaki T, Chatzi L. Incidence of parent reported food hypersensitivity in Greek children at 4 and 6 years of age: Results from a birth cohort study in Crete. *Clinical and Translational Allergy.* 2018;8(0).

33. Strinnholm A, Winberg A, West C, Hedman L, Ronmark E. Food hypersensitivity is common in Swedish schoolchildren, especially oral reactions to fruit and gastrointestinal reactions to milk. *Acta Paediatr.* 2014;103(12):1290-1296.

34. Topcu ZIK, Kaklikkaya N, Baki A, Orhan F. Characteristics of beef allergy in schoolchildren in Turkey. *Allergy and Asthma Proceedings* 2018;39(1):59-65.

35. Venkataraman D, Erlewyn-Lajeunesse M, Kurukulaaratchy RJ, et al. Prevalence and longitudinal trends of food allergy during childhood and adolescence: Results of the Isle of Wight Birth Cohort study. *Clin Exp Allergy.* 2018;48(4):394-402.

36. Westerlaken-Van Ginkel CD, Sprikkelman AB, Koppelman GH, Dubois AEJ, Vonk JM, Flokstra-De Blok BMJ. Likely questionnaire-diagnosed food allergy in 78, 890 adults from the northern Netherlands. *PLoS ONE* 2020;15(5):e0231818

37. Zuidmeer L, Goldhahn K, Rona RJ, et al. The prevalence of plant food allergies: a systematic review. *J Allergy Clin Immunol.* 2008;121(5):1210-1218.e1214.
